# Nucleolin Rescues TDP-43 Toxicity in Yeast and Human Cell Models

**DOI:** 10.3389/fncel.2021.625665

**Published:** 2021-04-12

**Authors:** Caterina Peggion, Maria Lina Massimino, Roberto Stella, Raissa Bortolotto, Jessica Agostini, Arianna Maldi, Geppo Sartori, Fiorella Tonello, Alessandro Bertoli, Raffaele Lopreiato

**Affiliations:** ^1^Department of Biomedical Sciences, University of Padova, Padova, Italy; ^2^CNR – Neuroscience Institute, Padova, Italy; ^3^Food Safety Division, Department of Chemistry, Istituto Zooprofilattico Sperimentale delle Venezie, Legnaro, Italy; ^4^Padova Neuroscience Center, University of Padova, Padova, Italy

**Keywords:** neurodegenerative disorders, TDP-43 proteinopathies, ALS, FTD, nucleolin, misfolded proteins, nucleo-cytoplasmic transport

## Abstract

TDP-43 is a nuclear protein involved in pivotal processes, extensively studied for its implication in neurodegenerative disorders. TDP-43 cytosolic inclusions are a common neuropathologic hallmark in amyotrophic lateral sclerosis (ALS) and related diseases, and it is now established that TDP-43 misfolding and aggregation play a key role in their etiopathology. TDP-43 neurotoxic mechanisms are not yet clarified, but the identification of proteins able to modulate TDP-43-mediated damage may be promising therapeutic targets for TDP-43 proteinopathies. Here we show by the use of refined yeast models that the nucleolar protein nucleolin (NCL) acts as a potent suppressor of TDP-43 toxicity, restoring cell viability. We provide evidence that NCL co-expression is able to alleviate TDP-43-induced damage also in human cells, further supporting its beneficial effects in a more consistent pathophysiological context. Presented data suggest that NCL could promote TDP-43 nuclear retention, reducing the formation of toxic cytosolic TDP-43 inclusions.

## Introduction

Neurodegenerative diseases (NDs) represent a huge health problem worldwide. In most cases, the origin of neurodegeneration remains largely unknown, but it is now commonly accepted that many of these disorders share the accumulation and aggregation of misfolded proteins as a major hallmark of neuropathology (for which they are often referred to as proteinopathies). Despite the role of such a protein misbehavior and the formation of protein assemblies may differ in distinct NDs, possibly also in different subtypes of the same ND, in all cases disease-associated misfolded proteins are supposed to either acquire toxic properties or lose their normal functions, or both, thereby driving neuropathogenic mechanisms (Ross and Poirier, 2004; Mathieu et al., 2020).

Among NDs, the vast majority of amyotrophic lateral sclerosis (ALS), frontotemporal dementia (FTD), and also a subset of late-onset Alzheimer’s disease (AD) cases are characterized by the presence of neuronal cytoplasmic inclusions mainly composed of an ubiquitinated and hyper-phosphorylated form of the transactive response DNA binding 43 kDa protein (TDP-43) (Gao et al., 2018). TDP-43 is an ubiquitously expressed RNA/DNA-binding protein, normally residing in the nucleus where it plays important functions in the regulation of gene expression by modulating transcription, transcript splicing, mRNA stability, and microRNA biogenesis (Buratti et al., 2001; Prasad et al., 2019). As a physiological response to a variety of stress conditions, TDP-43 may also shuttle from the nucleus to the cytosol, where – as other RNA-binding proteins – contributes to the formation of stress granules that regulate RNA metabolism and protein synthesis. The structural correlate of such a TDP-43 function is a low complexity C-terminal domain region that may undergo structural transitions and is critically involved in liquid-liquid phase separation and the assembly of membrane-less organelles (e.g., stress granules) (Sun and Chakrabartty, 2017). It is conceived, however, that when such a regulated, cell-protective mechanism fails or diverges to the formation of noxious protein condensation states, neurodegenerative cascades are activated (Wolozin, 2012). Intriguingly, the presence of structurally disordered regions is also shared by two other major ALS-related gene products [i.e., fused in sarcoma/translocated in liposarcoma (FUS/TLS) and C9orf72 dipeptide repeats (DPRs)], pinpointing structural transitions of disease-associated proteins as a major driving force toward neurodegeneration (Harrison and Shorter, 2017; Santamaria et al., 2017).

The above concept is further reinforced by acknowledging that intracellular TDP-43 aggregates are a major histopathological hallmark of ALS, found in post-mortem samples of 97% ALS patients (Scotter et al., 2015). Although there is no clear causal relationship between the formation of such aggregates and neuronal death, deregulated translocation of TDP-43 to the cytoplasm (Barmada et al., 2010) and TDP-43 propensity to undergo structural transitions and form liquid-like condensates (Bolognesi et al., 2019) seem both critical for disease pathogenesis. The biochemical nature of TDP-43 neuropathologic species (e.g., insoluble aggregates, liquid-like condensates), their cellular localization (e.g., nucleus, cytoplasm, membrane-less structures) and (loss of) function mechanisms are, however, still largely unknown. What is clearly established, on the contrary, is that most of the >50 mutations in the gene encoding TDP-43, found in 3–5% patients with familial ALS and at a minor extent in sporadic ALS cases (Kabashi et al., 2008; Sreedharan et al., 2008) promotes TDP-43 cytosolic translocation and aggregation/liquid-phase separation, and cytotoxicity, thus providing a strong link between such events and disease pathogenesis (Prasad et al., 2019).

A number of NDs associated to protein misfolding and aggregation, such as ALS, AD, Parkinson’s disease (PD), and Huntington’s disease (HD), have been successfully modeled in the yeast *Saccharomyces cerevisiae* (Ruetenik and Barrientos, 2018).

In the last decade, different yeast models have been generated ectopically expressing TDP-43 at either high or low levels, mainly by the use of multicopy or centromeric plasmids, respectively (Armakola et al., 2011). Integrative (single copy) expression systems have also been exploited (Johnson et al., 2008). Such studies suggested that also in yeast cells, the toxic effects of human TDP-43 were directly correlated to the protein expression levels (Johnson et al., 2008; Armakola et al., 2011), as already demonstrated in animal and neuronal disease models (Barmada et al., 2010, 2014; Baloh, 2011). In the attempt to better control TDP-43 levels due to ectopic expression systems (Karim et al., 2013), we here generated novel yeast models that conditionally express human TDP-43 (either WT or bearing ALS-related mutations), from either single or multiple copies of the coding sequence stably integrated in the yeast genome by the CRISPR-Cas9 technique. The main purpose of this strategy was the generation of yeast models for TDP-43 proteinopathies expressing the protein at finely tuned levels that may be optimally suited for the functional analysis of potential modulators of the TDP-43 cytotoxicity.

Different large-scale approaches in yeast and other cell paradigms (*Drosophila* and human cells) supported the linkage between ALS/FTD and the function of intra- and extra-nuclear membrane-less organelles, identifying some nucleolar proteins, cytosolic stress granules components and nucleocytoplasmic trafficking factors as genetic modifiers (i.e., suppressors or enhancers) of the cytotoxicity triggered by ALS-related proteins, such as C9orf72 DPRs (Jovičič et al., 2015; Lee et al., 2016). Moreover, some nucleolar proteins have been identified as TDP-43 interactors in systematic mass spectrometry (MS)-based approach in mammalian cells (Freibaum et al., 2010).

Among these proteins, we focused on nucleolin (NCL) in light of multiple aspects, including the following structural and functional features of the protein that are reminiscent of TDP-43 attributes: (i), NCL is able to bind DNA/RNA molecules thanks to multiple RNA recognition motifs (RRM) domains; (ii), NCL carries an intrinsically disordered C-terminal region able to interact with different functional partners; (iii) NCL is one of the most abundant non-ribosomal proteins in the nucleolus, where it plays fundamental roles in nucleolar assembly and function, rRNA and ribosome metabolism (Jia et al., 2017), which are critically perturbed in ALS and FTD (Lehmkuhl and Zarnescu, 2018). Noteworthy, although mainly localized in the nucleolus, NCL is able to shuttle in the cytosol (and to the plasma membrane) where it contributes to the transport of RNA molecules and ribosomal proteins, playing roles in both nucleocytoplasmic trafficking and several signaling pathways, such as cell proliferation and survival (Jia et al., 2017).

We are here providing evidence that NCL is able to act as a potent suppressor of TDP-43 toxicity, relieving the lethal damage induced by WT or ALS-mutant TDP-43 overexpression in yeast cells. Additional experiments in mammalian HEK293T cell models further support that the NCL overexpression can prevent TDP-43-dependent toxicity, reducing the formation of detergent-insoluble assemblies and restoring cell viability, possibly by avoiding the non-physiologic translocation of TDP-43 out of the nuclear compartment.

## Materials and Methods

### Yeast Strains and Plasmids

All yeast strains and plasmids used in this study are listed in Supplementary Tables 1, 2, respectively. Methods to generate yeast strains and plasmids are reported in the Supplementary Methods section. Primers used are listed in Supplementary Tables 3–6.

### Yeast Media, Transformation and Yeast Cell Viability Assays

Procedures for yeast growth and manipulation were performed according to standard protocols (Amberg et al., 2005; Vokes and Carpenter, 2008).

Yeast cells were grown in standard rich media (10 g/L Bacto-yeast extract, 20 g/L Bacto-peptone) supplemented with 20 g/L of glucose or galactose as carbon source, or in synthetic minimal media (1.7 g/L yeast nitrogen base without amino acids, 5 g/L ammonium sulfate) containing 20 g/L of glucose, raffinose or galactose and lacking the specific nutrients allowing for selection of transformed yeast clones. Media components were from Difco (Thermo Fischer Scientific), and auxotrophic requirements were from Sigma-Aldrich. Induction of gene expression under Gal1 promoter control was obtained by growing transformed yeast cells in raffinose containing medium for 16 h, followed by dilution of the cultures to an optical density at 600 nm light adsorption (OD_600_) of 0.1 and growth in galactose containing medium. Cells were routinely incubated at 30°C.

The PEG/lithium acetate method was used to transform yeast (Gietz and Woods, 2006) and then cells were plated on selective solid media.

Yeast cell viability was performed as previously described (Mirisola et al., 2014) by measuring the ability of yeast cultures to grow on solid (spotting assays) and liquid media (OD_600_ culture turbidity measurements). In particular, for spotting assays, yeast cells were grown overnight at 30°C in liquid media containing raffinose. Cultures were then normalized to OD_600_ = 1, serially diluted and spotted onto synthetic solid media containing glucose or galactose and the auxotrophic requirements as needed. Plates were maintained at 30°C for different days, as indicated. Conversely, OD_600_ measurements were performed in a Microplate Reader spectrophotometer (TECAN) at a 600 nm wavelength (bandwidth 9 nm) with 15 flashes and 200 μL of culture per well. Briefly, three yeast clones were grown overnight in a raffinose containing medium (non-inducing condition), diluted in triplicate to OD_600_ = 0.1 in a galactose containing medium (inducing condition), and grown for 24 h. OD_600_ was then recorded immediately after the transfer (*t*_0_) or after 24 h of growth (*t*_24_), in the galactose medium, and the OD_600_ ratio (*t*_24_/*t*_0_) was calculated. Data were then normalized to the mean ratio of control cultures CEN.PK IMX672 (hereafter abbreviated as CENPK).

### *S. cerevisiae* Imaging

For fluorescence microscopy observations, cells grown for 6 h in a galactose-containing liquid medium were used, while for cell shape analysis by brightfield microscopy cells were cultured on galactose containing solid substrate for 2 days. In both cases, before microscopy analysis, harvested cells were washed and resuspended in PBS, and then mounted on a coverslip with a thin agar slab as described previously (Skinner et al., 2013). Images were acquired using an inverted microscope (CTR6000, Leica) equipped with a Xenon lamp, a suited fluorescence excitation/dichroic/emission filter setting (λ_*ex*_ = 488 nm for the excitation of GFP) and a computer-assisted charge-coupled device camera (Hamamatsu Orca flash 4.0), which allowed the acquisition of digital micrographs for either fluorescence or brightfield microscopy.

For morphometric measurements, the cell length-to-width ratio was calculated on digitalized images using the Fiji ImageJ software.

For confocal microscopy observations, 1 mL of yeast cultures grown 24 h in galactose-containing medium were fixed in 4% formaldehyde (15 min, RT). To stain nuclei, DAPI (Sigma-Aldrich, 2 μg/mL final concentration) was added during cell fixation. After fixation, cells were washed twice with sterile water and then resuspended in 1 M sorbitol. Cell suspension was dropped onto a slide (Superfrost, BDH) and covered with a coverslip (Noree and Sirinonthanawech, 2020). Cells were observed with a Leica SP5 confocal microscope using 63X HCX PL APO (NA 1.4) oil-immersion objectives. Laser excitation line, power intensity, and emission range were chosen accordingly to fluorophore in different samples to minimize bleed-through. During acquisition, parameters for laser intensity and photomultiplier gain were kept constant. Imaging was performed at 1024 × 1024 pixels, with a 200 Hz acquisition rate, by capturing Z-series that covered the entire field of interest. Every planes of a Z-stack scan acquisition were merged into a single image using Fiji/ImageJ software.

### Whole Protein Extraction From *S. cerevisiae* Cells

The extraction of proteins from yeast cells for Western blot (WB) and MS analyses was performed as described elsewhere (Kushnirov, 2000) with some modifications. Briefly, yeast cells were harvested by centrifugation (6,000 × *g*, 10 min, 4°C). Cells were then resuspended in 0.1 M NaOH and incubated at room temperature for 20 min. After pelleting, cells were finally lysed by vortexing (2 min) in a buffer containing 62.5 mM Tris-HCl (pH 6.8), 2.3% (w/v) SDS and 10% (w/v) glycerol (buffer O). Lysates were boiled (3 min) and, after centrifugation to remove cell debris, protein quantification was performed in the supernatant using a bicinchoninic acid (BCA) assay kit (Thermo Fisher Scientific), according to the manufacturer’s instructions.

### Yeast Sample Preparation for Tandem Mass Tag-Based Quantitative Proteomics

For tandem mass tag (TMT)-based quantitative proteomic analysis, protein extracts (50 μg) from three biological replicates of parental CENPK and TDP 1C yeast strains were processed according to the filter-aided sample preparation method (Wisniewski et al., 2009), using filters with 10,000 Da as molecular weight cut-off (Sartorius). Briefly, filters were washed four times with 8 M urea and 100 mM triethylammonium bicarbonate (TEAB) buffer (Sigma-Aldrich) and then treated in 100 mM TEAB containing 25 mM dithiothreitol (DTT) (45 min, 55°C) to reduce disulfide bonds, and then in the same buffer (100 mM TEAB) containing 55 mM iodoacetamide (45 min in the dark, RT) to block reduced cysteine residues. Finally, filters were washed twice using 100 mM TEAB and, afterward, protein digestion was performed by adding 100 μL of sequencing grade modified trypsin (20 μg/mL^–1^ in 100 mM TEAB, pH 8.0) to each filter (18 h, 37°C). After digestion, filters were subjected to two subsequent centrifugation step (14,000 × *g*, 10 min) adding 50 μL of 100 mM TEAB for peptide recovery. Peptide mixtures were then labeled using 6-plex TMT reagents (Thermo Scientific), and subjected to fractionation and purification as previously described (Biancotto et al., 2019). The three biological replicates of CENPK samples were labeled with the 126.1 Th, 127.1 Th, and 128.1 Th TMT mass tags, respectively, while the three TDP 1C samples were labeled with the 129.1 Th, 130.1 Th, and 131.1 Th TMT mass tags, respectively. After labeling, the six samples were mixed in equal total protein amounts and subjected to a desalting step by means of pre-activated and pre-equilibrated C_18_ BioPure spin columns (The Nest Group). To reduce sample complexity, TMT-labeled peptides were fractionated by strong cation exchange fractionation (SCX) before liquid chromatography (LC) and MS. Briefly, labeled samples were diluted with four volumes of 0.1% formic acid and then applied to the SCX macro spin columns (The Nest Group). After two washings, retained peptides were stepwise eluted in five fractions containing increasing concentrations of ammonium formate (i.e., 100, 200, 300, 400, 500 mM). Prior to sample injection into the high-performance liquid chromatography-high resolution tandem mass spectrometry (HPLC-HRMS/MS) workstation, each fraction was desalted by means of the C_18_ BioPure spin columns. The five labeled fractions of each peptide extract were finally dried using a stream of nitrogen (RT) and suspended in 100 μL of 0.1% formic acid for the subsequent MS analysis.

### HPLC-HRMS/MS Analysis

Relative quantification of yeast proteins was achieved using a hybrid quadrupole-orbitrap Q-Exactive (Thermo Fisher Scientific) mass spectrometer, coupled to a UHPLC system Ultimate 3000 (Thermo Fisher Scientific). Each SCX fraction was separated on a reverse-phase analytical column (Aeris peptide C_18_, 150 mm × 2.1 mm, 2.6 μm, Phenomenex) kept at 30°C.

Elution solvents for peptide separation were water (A) and acetonitrile (ACN) (B), both containing 0.1% (v/v) formic acid. The chromatographic separation was carried out at a flow rate of 200 μL × min^–1^ (35 min) using a gradient elution with the following composition (expressed as A:B ratio): 97.5:2.5 for 1 min; from 97.5:2.5 to 70:30 in 19 min (following a linear gradient), from 70:30 to 50:50 in 4 min; from 50:50 to 5:95 in 2 min and maintained for 4 min to wash the column; then back to 97.5:2.5 (to re-equilibrate the system) in 0.5 min and maintained for 4.5 min. The injection volume was 10 μL.

Ion source capillary temperature was 325°C, the sheath gas flow rate 35 (arbitrary instrument units), auxiliary gas flow rate 10 (arbitrary instrument units), S-lens voltage 55 V, heater temperature 325°C, and the spray was optimized at 3 kV. The instrument operated in data-dependent mode with a top-7 acquisition method (i.e., a full MS scan at 70,000 resolution on the orbitrap, followed by the MS/MS fragmentation of the seven most intense ions). Full scan spectra were acquired using an automatic gain control (AGC) target of 3 × 10^6^ ions, an injection time (IT) of 250 ms, an isolation window of 2.0 Th, and a scan range from 300 to 2000 Th. Higher energy C-trap dissociation (HCD) was performed with a normalized collision energy of 30, AGC target of 2 × 10^5^ ions, an IT of 120 ms, a dynamic exclusion of 30 s and a resolution of 17,500. Fragmentation spectra were used for peptide identification and quantification, setting a fixed starting mass of 100 Th. To increase the number of identified peptides, each SCX fraction was analyzed twice. To this purpose, the m/z values of peptides positively identified in the first analysis (as described in details in the next section) were used to create a static exclusion list that was then applied to a second HPLC-HRMS/MS analysis (under the same chromatographic and instrumental conditions) for each sample fraction.

### Untargeted MS Data Analysis

Raw files derived from HPLC-HRMS/MS runs were analyzed with a MudPIT protocol using the Proteome Discoverer 1.4 software (Thermo Fisher Scientific) interfaced to a SEQUEST HT search engine (Thermo Fisher Scientific). All MS/MS data were searched against the UniProt *S. cerevisiae* database (3AUP000002311). Enzyme specificity was set to trypsin, and a maximum of one missed cleavage was allowed. The precursor and product ion mass tolerances were set to 10 ppm and 0.2 Da, respectively. Oxidation of methionine was selected as variable modification, while 6-plex TMT at N-termini and at lysine residues, and carbamidomethylation of cysteines were set as fixed modification. Relative quantification was performed directly by the Proteome Discoverer software and only unique peptides were considered for quantification purposes. Based on the Percolator algorithm, proteins were considered as correctly identified if at least two unique peptides were quantified with an individual *q*-value < 0.05. Proteins were then grouped according to the principle of maximum parsimony. For quantification, the reporter mass tolerance was set to 20 ppm. The software detected the reporter ions (126.1, 127.1, 128.1, 129.1, 130.1, 131.1 Th), and performed the quantification of relative peptide abundance normalizing the intensity of each reporter ion to that of CENPK 1 sample (126.1 Th). Normalized intensity values of proteins derived from Proteome Discoverer were exported in an Excel spreadsheet, and the matrix was arranged for further analysis. The final fold-change of a given protein was calculated as the mean value of the normalized ratios (TDP 1C/CENPK) of the three replicates. Finally, a two-tailed *t*-test was performed, and only proteins with a ratio > 1.33 or <0.77, and a *p*-value < 0.05, were considered over-expressed or under-expressed, respectively.

### Parallel Reaction Monitoring

The targeted MS-based parallel reaction monitoring (PRM) analysis was carried out in WT CENPK, and TDP-43 1C and 2C strains expressing, or not, NCL. To this end, 50 μg of yeast lysates from nine different cultures for WT CENPK and for each of the four different TDP-43 clones (for a total 45 samples) were prepared and processed according to the filter-aided sample preparation (FASP) method as described above. After digestion, peptide mixtures were acidified (pH < 3) by adding formic acid and desalted using BioPure C_18_ spin columns (The Nest Group), following manufacturer’s instructions. Briefly, samples were loaded in pre-activated C_18_ spin columns, that were washed twice with 200 μL of 0.5% formic acid (v/v), and then peptides were eluted using 75% ACN containing 0.1% formic acid (v/v). Peptide extracts were dried under a stream of nitrogen and, immediately before HPLC-HRMS/MS analyses, dissolved in a solution (100 μL) containing 5% (v/v) ACN and 0.1% (v/v) formic acid to obtain a final concentration of 0.5 μg × μL^–1^ of protein digest. Confirmatory analyses were performed using the same HPLC-HRMS/MS apparatus described above operating in the PRM mode. Peptides were separated using the same chromatographic gradient described above. The scheduled PRM method was developed by recording and selecting the most intense charge state of the considered peptides and four diagnostic precursor-to-product ion transitions in serial injections of a representative yeast digest sample. The same was done for glyceraldehyde 3-phosphate dehydrogenase (GAPDH), which was chosen as housekeeping protein. PRM acquisition parameters were as follows: HCD fragmentation with normalized collision energy (NCE) of 27, AGC target of 5 × 10^5^ ions, an IT of 120 ms, an isolation window of 1.6 Th with an offset of 0.4 Th. Two proteotypic peptides were selected for each protein included in the PRM analysis. The selected precursor-to-product ion transitions, together with the retention time and the instrumental settings used in the acquisition method are reported in Supplementary Table 7.

To ensure that no instrumental drift occurred during PRM analysis, a pooled sample prepared by mixing together equal amounts of peptide digests was injected and analyzed at the beginning and at the end of the analytical sequence. The Skyline software (version 3.5.0.9191) (MacLean et al., 2010) was used to assess the relative abundance of each peptide by calculating the total peak area under the curve (AUC) of the chromatographic peaks deriving from four precursor-to-product ion transitions recorded for target peptides. Each AUC value in the different samples was normalized to the AUC of the corresponding peptide calculated in the pooled sample. Protein abundance was then calculated by averaging the normalized peptide values calculated for the two monitored peptides. Then protein abundance values were further normalized to the GAPDH abundance calculated for each sample to obtain relative protein abundance values to be compared between WT CENPK and the different TDP-43 yeast strains (with or without NCL).

The MS proteomics data have been deposited to the ProteomeXchange Consortium via the PRIDE partner repository (Vizcaíno et al., 2009) with the dataset identifier PXD022432.

### HEK293T Cell Culture and Transfection

Human epithelial kidney HEK293T cells were grown in Dulbecco’s modified Eagle medium (DMEM) supplemented with 10% (v/v) foetal bovine serum (FBS), 2 mM L-glutamine, 100 U/mL penicillin, and 100 μg/mL streptomycin and maintained at 37°C in a humidified incubator with 5% CO_2_. HEK293T cells were seeded at 25% of confluence in multi-well culture plates 24 h before transfection. Cells were (co-)transfected using the Lipofectamine 3000 transfection kit (Invitrogen) following the manufacturer’s instructions and analyzed 48 h after transfection. Plasmids used to transfect HEK293T cells are reported in Supplementary Table 2. Details regarding the construction of plasmids are described in the Supplementary Methods section. Generally, we obtained 40% of co-trasfection efficiency, determined by calculating the percentage of GFP and mKATE2 positive cells in the total population (Hoechst stained).

### HEK293T Cell Viability Assay

Cell viability was assessed using the MTS [3-(4,5-dimethylthiazol-2-yl)-5-(3-carboxymethoxy-phenyl)-2-(4-sulfo phenyl)-2H-tetrazolium, inner salt] assay (CellTiter96 Aqueous One 5 Solution Assay, Promega), based on the reduction of MTS by viable cells, following the manufacturer’s instructions. Briefly, cells were seeded in 96-wells and co-transfected with the desired plasmids (as indicated in the figure legends), and – 48 h after transfection – the cell culture medium was removed and the MTS reagent [20 μL in 100 μL of phosphate-buffered saline (PBS)] was added to each culture well. Cells were then incubated at 37°C (90 min), after which the absorbance of reduced MTS (λ = 490) was determined using a Microplate Reader (TECAN) spectrophotometer.

### Confocal Microscopy

For confocal microscopy observations, HEK293T cells were seeded onto 13 mm coverslips and co-transfected using plasmids coding for the desired fluorescent constructs. 48 h after transfection, cells were rinsed twice with PBS, fixed (30 min, 4°C) with paraformaldehyde [2% (w/v) in PBS], rinsed again and permeabilized (15 min, 4°C) with Triton-X100 [0.1% (w/v) in PBS]. Cell nuclei were counter-stained with Hoechst 33342 [(5 μg/mL in PBS) 10 min, RT, Sigma-Aldrich], and coverslips were finally washed in PBS and mounted in Mowiol 40–88 (Sigma-Aldrich) [8% (w/v) in glycerol:PBS (1:3, v/v)]. Images were collected with a Leica SP5 confocal microscope using 40X or 63X HCX PL APO (NA 1.25 or 1.4, respectively) oil-immersion objectives. Settings of confocal microscope and image acquisition and analysis were performed as described above. For quantitative analyses reported in Figure 9, six different fields from three independent biological replicates were used.

### HEK293T Cell Lysis and Protein Extraction

For protein extraction, HEK293T cells, grown onto 12-wells plates and transfected as described above, were washed twice with ice-cold PBS and lysed with buffer O (60 μL/well). After centrifugation (14,000 × *g*, 10 min, 4°C) to precipitate cell debris, the total protein content in the supernatant was determined by the BCA assay kit (Thermo Fisher Scientific).

### Western Blot and Antibodies

For WB analyses, 10–20 μg of proteins diluted in reducing sample buffer [buffer O added with 50 mM DTT and 0.01% (w/v) bromophenol blue] were subjected to SDS-PAGE [using 10% (w/v) acrylamide-*N,N’*-methylenebisacrylamide [37.5:1 (w/w)] or Mini-Protean TGX precast gels (4–15%, Bio-Rad Laboratories)] and electroblotted onto polyvinylidene difluoride (PVDF) membranes (0.45 μm pore size; Bio-Rad Laboratories). Membranes were stained with Coomassie brilliant blue (Sigma-Aldrich) to check for even protein loading, and digitalized images were collected for subsequent densitometric analysis. After Coomassie destaining with methanol, membranes were incubated (1 h, RT) in a blocking solution[5% (w/v) non-fat dry milk (Bio-Rad Laboratories) in TRIS-buffered saline (TBS) added with 0.1% (w/v) Tween-20 (TBS-T)], and then probed (overnight, 4°C) with the desired primary antibody diluted in TBS-T containing 1% (w/v) bovine serum albumin (BSA). After three washings with TBS-T, membranes were incubated (1 h, RT) with horseradish peroxidase-conjugated anti-mouse or anti-rabbit IgG secondary antibody (Sigma-Aldrich, cat. nos. A9044 and A0545, respectively) (1:60,000 in blocking solution), depending on the primary antibody. Immunoreactive bands were visualized using an enhance chemiluminescence reagent kit (EMD Millipore) and digitalized by means of an UVItec imaging system (Eppendorf). For densitometric analyses, the intensity of each immunoreactive band was normalized to the optical density of the corresponding Coomassie blue-stained lane (Colella et al., 2012).

The following primary antibodies (Abs) were used (dilutions in parentheses): anti-TDP-43 mouse monoclonal (m)Ab (1:1,000, Santa Cruz Biotechnology, cat. no. sc-376532); anti-NCL rabbit polyclonal (p) Ab (1:1,000; Santa Cruz Biotechnology, cat. no. sc-13057); anti GFP mouse mAb (1:2,000, Roche, cat. no.11814460001); anti RFP mouse mAb (1:5,000, Abcam, cat. no. 62341); anti BCL2 rabbit pAb (1:1,000, Sigma-Aldrich, cat. no. SAB4500003); anti caspase 3 mouse mAb (1:1,000, Santa Cruz Biotechnology, cat. no. sc-56052); anti caspase 9 rabbit pAb (1:1,000, Sigma-Aldrich, cat. no. SAB4300683).

### Protein Co-immunoprecipitation From *S. cerevisiae* and HEK293T Cell Lysates

For protein co-immunoprecipitation (co-IP) assays from *S. cerevisiae*, 10 mL of the TDP 2C yeast strain transformed with the NCL-mKATE2-pYES2 construct was grown for 8 h (reaching OD_600_ ∼ 1) in galactose-rich medium, harvested by centrifugation (6,000 × *g*, 5 min) and resuspended in IP buffer [25 mM Tris-HCl (pH 7), 75 mM NaCl, 1 mM EDTA, 2.5% (w/v) glycerol, 0.2% (w/v) NP-40, 0.5 mM DTT, 1 mM PMSF and complete EDTA-free protease inhibitor cocktail (Roche)]. Cells were lysed by vortexing (30 s, 6000 rpm) in a MagnaLyser apparatus (Roche Diagnostics). The protein fraction was obtained after precipitation of cell debris by centrifugation (14,000 × g, 10 min, 4°C), and proteins were quantified by a Lowry assay kit (Sigma-Aldrich). 1 mg of total proteins (in IP buffer) was added with either 1 μg of anti-NCL rabbit pAb (Santa Cruz Biotechnology, cat. no. sc-13057), or anti-GFP mouse mAb (Roche, cat. no. 11814460001), or anti-RFP mouse mAb (Abcam, cat. no. 62341) antibody and incubated (16 h, 4°C) under continuous gentle inversion mixing. As negative control, the same amount of lysate was incubated in the absence of antibody.

The co-IP protocol for HEK293T cells was adapted from Freibaum et al. (2010) with minor modifications. Briefly, cells co-transfected with the desired plasmids were resuspended in ice-cold lysis buffer (137 mM NaCl, 2.7 mM KCl, 8 mM Na_2_HPO_4_, and 2 mM KH_2_PO_4_, 0.2% NP-40, 10% glycerol (w/v), 5 mM EDTA and Roche complete EDTA-free protease inhibitor cocktail), and maintained on ice for 15 min. Cell lysates were then passed (five times) through 21-gauge needles and centrifuged (20,000 × *g*, 4°C, 15 min). The total protein content in the supernatant was determined using the BCA assay kit (ThermoFisher Scientific), and 200 μg of total proteins were incubated (16 h, 4°C) with 0.2 μg of the rabbit polyclonal anti-NCL (Santa Cruz Biotechnology, cat. no. sc-13057) in lysis buffer under continuous gentle inversion mixing. When needed, cell lysates were treated with RNAse A (Thermo Fisher, cat. no. EN0531, 2 μg, 4°C, 10 min) prior to immunoprecipitation. Non-transfected cells were processed as described above as negative control.

For both yeast and HEK293T samples, protein-antibody complexes were precipitated by adding protein A-Sepharose (2 mg, Sigma-Aldrich, cat. no. P3391). After incubation (1 h, 4°C) under gentle shaking, sepharose bead-bound immunocomplexes were collected by centrifugation (3,000 × *g*, 4°C), washed three times with lysis buffer and once with 50 mM Tris–HCl (pH 7.5), and finally boiled (3 min) in reducing sample buffer. Immunoprecipitated proteins were separated onto 10% SDS-PAGE gel, electroblotted onto PVDF membranes (Millipore) and analyzed by WB with antibodies to target proteins.

### Detergent-Solubility Assays in *S. cerevisiae* and HEK293T Protein Extracts

The protocol for detergent-soluble and -insoluble protein fractionation from yeast cells was adapted from Alberti et al. (2010). Briefly, 10 mL of yeast cultures grown for 6 h (reaching OD600 ∼ 0.8-1) in inducing (galactose-rich) medium were harvested by centrifugation, washed with water and pelleted again. The cell pellet was resuspended in 500 μL of lysis buffer [25 mM Tris-HCl (pH 7.5), 75 mM NaCl, 1 mM EDTA, 2.5% glycerol (w/v), 0.5% Triton X-100 (v/v), 0.25% deoxycholate (w/v), 0.05% SDS (w/v), 0.5 mM DTT, 1 mM PMSF and complete EDTA-free protease inhibitor cocktail (Roche)]. Cells were lysed by vortexing (30 s × 6 times, 6,000 rpm) in a MagnaLyser apparatus (Roche Diagnostics), and a small fraction of the crude lysate (“total” fraction) was kept for subsequent analysis. The remaining crude lysate was centrifuged (800 × *g*, 5 min, 4°C) to precipitate cell debris. Fractionation was performed by centrifuging (100,000 × *g*, 30 min, 4°C) the cleared lysate, the supernatant (“detergent-soluble” fraction) was saved, and the pellet was resuspended in lysis buffer and centrifuged again as above. The final pellet (“detergent-insoluble” fraction) was resuspended in a urea-based buffer containing 7 M urea, 2 M thiourea, 4% CHAPS and 30 mM Tris (pH 8.5).

Detergent solubility fractionation of proteins from HEK293T cells was performed according to Cohen et al. (2012) with some modifications. Cells grown in 6-well plates and transfected as previously described were washed in PBS and harvested by centrifugation (2,000 × *g*, 5 min, 4°C). Cells were then resuspended in 300 μL of radio-immunoprecipitation assay (RIPA) buffer [20 mM Tris-HCl (pH 7.5), 150 mM NaCl, 1% NP-40 (v/v), 0.1% sodium deoxycholate (w/v), 1 mM Na_3_VO_4_] supplemented with 2 mM EDTA, 1 mM EGTA and complete EDTA-free protease inhibitor cocktail (Roche). Following incubation on ice (15 min), cells were sonicated (10 s) and the obtained lysate was cleared by centrifugation (500 × *g*, 10 min) to remove cell debris. A small amount of the lysate (“total” fraction) was kept for subsequent analysis, while the remaining lysate was centrifuged (100,000 × *g*, 30 min, 4°C). The resulting supernatant (“detergent-soluble” fraction) was saved and the pellet, after washing in RIPA buffer, was centrifuged again as above. The final pellet (“detergent-insoluble” fraction) was resuspended in the same urea-based buffer used for yeast samples.

The quantification of the protein content in all fractions of both yeast and HEK293T samples was carried out by the Lowry assay kit (Sigma-Aldrich). Protein fractions were then analyzed by SDS/PAGE [using 10% (w/v) acrylamide-*N,N’*-methylenebisacrylamide 37.5:1 (w/w)] and WB for the detection of the target proteins as described previously. To determine the amount of RIPA-soluble (or insoluble) TDP-43, the percentage ratio between the intensity of TDP-43 soluble (or insoluble) immunoreactive bands and the sum of the intensity of TDP-43 bands in the soluble and insoluble fractions were calculated. The reliability of the fractionation protocol was confirmed by evaluating the absence of GAPDH in the insoluble fractions.

### Computational Biology

The protein–protein interaction network and gene ontology enrichment analysis was performed using the publicly available Cytoscape 3.7 software (Shannon et al., 2003) integrated with the STRING application (Doncheva et al., 2019). The STRING database uses a combination of prediction approaches integrated with other information (i.e., neighborhood, transferred neighborhood, gene fusion, co-occurrence, co-expression, experimental data, databases, text mining). The protein network was built using default parameters (minimum required interaction score = 0.4), including all active prediction parameters.

### Statistical Analysis

Data were analyzed using Prism 7 (GraphPad Software) and Microsoft Excel (Microsoft Corporation) software. Data are reported as mean ± standard error of the mean (SEM). The number of biological replicates (n) reported in figure legends indicates the number of cultures/transformations, or the number of transfection experiments performed in different days, for yeast and HEK293T cells, respectively. Depending on the experiment, the statistical analysis was carried out as indicated in each figure legend. A *p*-value < 0.05 was considered statistically significant (^∗^*p* < 0.05, ^∗∗^,^•^ *p* < 0.01, ^∗∗∗^*p* < 0.001, ^****^, ■ *p* < 0.0001).

## Results

### Generation and Characterization of Novel Yeast Models for TDP-43 Proteinopathies

Since one chromosomally integrated copy of TDP-43 displayed no toxicity to yeast cells (Johnson et al., 2008), we generated (by CRISPR/Cas9 genome editing) novel *S. cerevisiae* yeast strains in the CEN.PK IMX672 genetic background (Mans et al., 2015; hereafter abbreviated as CENPK), carrying in the yeast genome two (2C) or three (3C) integrated copies of the wild-type (WT) human TDP-43 sequence fused to GFP. To note that, as already demonstrated, the GFP-tagging did not affect the TDP-43-related toxicity in yeast (Johnson et al., 2008). In order to prevent cell toxicity during normal cell growth, we placed the TDP-43-GFP transgene under the control of the tightly regulated galactose-inducible *GAL1* promoter, and inserted it into the yeast genome by replacing auxotrophic non-functional alleles. As controls, we also generated CENPK yeast strains carrying one copy of the TDP-43-GFP chimera integrated into the genome (1C), or strongly overexpressing the protein by means of an ectopic galactose-inducible multi-copy (MC) plasmid. The genome editing correctness in the obtained yeast strains was verified by PCR (Supplementary Figure 1). The expression of TDP-43 in the different yeast models at different time points after galactose induction was assessed by WB analysis, which confirmed that the protein expression levels were time- and dose/copy-dependent (Supplementary Figure 2).

**FIGURE 1:**
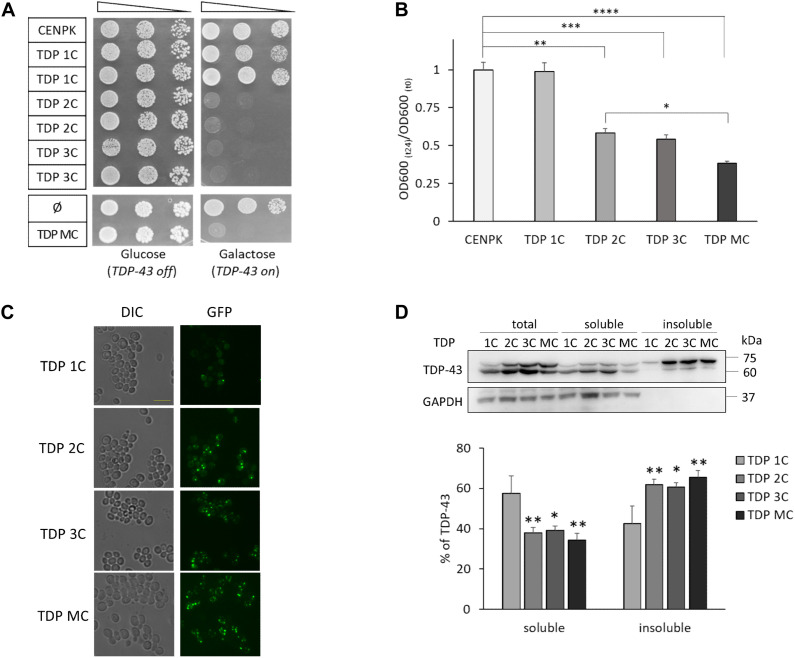
Two or more copies of the human TDP-43 transgene are lethal to *S. cerevisiae* cells, with increasing levels of TDP-43 in detergent-insoluble fraction. **(A)** The viability of the unmodified *S. cerevisiae* strain (CENPK) was compared to yeast strains carrying in the genome one (TDP 1C), two (TDP 2C), or three (TDP 3C) copies of the wild-type (WT) TDP-43 transgene (upper panel) or transformed with either non-integrative multi-copy plasmid empty (ø), or coding for human TDP-43 (TDP MC) (lower panel). Yeast cells (OD_600_ = 1) of each strain were serially diluted (10-fold) and spotted onto either inducing medium plates (galactose, TDP-43 on), or repressing condition as control (glucose, TDP-43 off), and incubated at 30°C for 3 days. Data are representative of three experiments yielding comparable results. **(B)** After inducing TDP-43 expression in galactose-containing liquid medium (24 h, 30°C), the growth rate of the above yeast strains was measured as the ratio of the OD_600_ at the end (*t*_24_) and the beginning (*t*_0_) of induction [OD_600_ (*t*_24_)/OD_600_ (*t*_0_)]. Data [mean ± standard error of the mean (SEM)] are reported after normalization to the mean value of the CENPK strain. *n* = 12, ^∗^*p* < 0.05, ^∗∗^*p* < 0.01, ^∗∗∗^*p* < 0.001, ^*⁣*⁣**^p < 0.0001 Kruskal–Wallis test followed by a Dunn’s *post hoc* test. **(C)** After TDP-43 induction in galactose-containing liquid medium (6 h, 30°C), cells of the different yeast strains were observed by epifluorescence microscopy (GFP) to visualize the expression of the TDP-43-GFP fusion protein. For each fluorescence image, differential interference contrast (DIC) micrograph is also reported. Images are representative of three biological replicates for each strain. *Scale bar*, 10 μm. **(D)** RIPA-lysates from yeast strains, harboring the indicated copies of the TDP-43 transgene, were subjected to biochemical separation of detergent-soluble and -insoluble protein fractions. Total, soluble and insoluble fractions (1:1:1 ratio) were analyzed by WB for the presence of TDP-43 or GAPDH as a control for RIPA-soluble proteins (upper panel). The lower panel reports the densitometric analysis of TDP-43-immunoreactive bands in RIPA-soluble and -insoluble fractions quantified as described in Materials and Methods. Here and after, data are reported as mean ± SEM, *n* = 3 (here and after indicating biological replicates), ^∗^*p* < 0.05, ^∗∗^*p* < 0.01, one way ANOVA with Sidak correction for comparison of TDP 2C, TDP 3C and TDP MC to TDP 1C.

Firstly, we performed functional assays aimed at investigating the effects of TDP-43 expression on yeast viability. As shown in the spot test reported in Figure 1A, the survival of yeast cells was unaffected in glucose-containing medium (TDP-43 expression off), whereas cells carrying two (or more) copies, but not one copy, of TDP-43 died when grown in galactose-containing medium (TDP-43 expression on). We further monitored the growth rate in liquid cultures of yeast strains carrying a different copy number of the TDP-43 construct (1C, 2C, 3C, and MC) after 24 h of galactose induction compared to the CENPK control strain. As shown in Figure 1B, while a single TDP-43 copy did not significantly affect yeast growth, the detrimental effects of TDP-43 on the growth rate increased with increasing the transgene copy number. Collectively taken, the above data indicate that TDP-43 cytotoxicity in the genetically engineered yeast strains correlated with TDP-43 copy number, thus to the extent of TDP-43 expression during the first hours after galactose induction (Supplementary Figure 2).

Taking advantage from the GFP tag in the chimeric TDP-43 construct, we also visualized its intracellular distribution in yeast cells by fluorescence microscopy. As shown in Figure 1C, TDP-43 fusion protein localized in discrete foci in all TDP-43-expressing yeast strains soon after induction (6 h). Our observation is consistent with previous findings reporting that yeast cells expressing TDP-43 (by one or more alleles) may contain a variable number of inclusion foci, which are also different in size and widely dispersed within the cells (Park et al., 2017; Bolognesi et al., 2019).

We then addressed more precisely the issue of TDP-43 aggregation by biochemical separation of detergent-soluble and -insoluble fractions from total protein extracts, as previously described (Johnson et al., 2009). Data reported in Figure 1D showed that, after 6 h of induction, the majority of TDP-43 (∼60%) was detected in the insoluble fraction of all yeast strains carrying two or more copies of the transgene, contrary to what observed in the single-copy strain (in which only the ∼40% of TDP-43 was found in the insoluble fraction), thereby correlating the formation of detergent-insoluble TDP-43 aggregates to the toxic effects of the protein.

**FIGURE 2:**
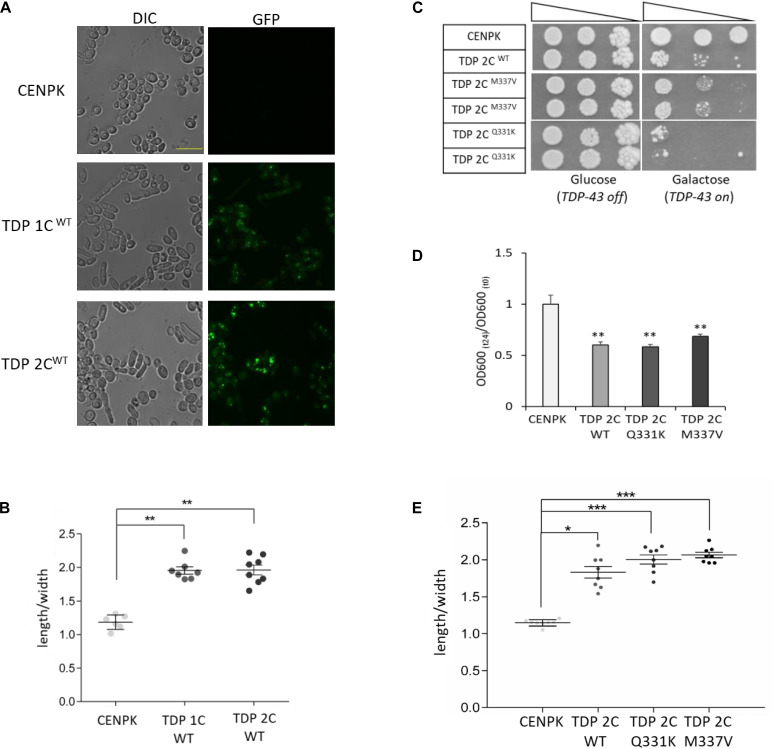
WT and ALS-associated mutants of TDP-43 alter the morphology of yeast cells from rounded to elongated. **(A)** Yeast cells carrying one (1C) or two (2C) copies of WT TDP-43 transgene, and the CENPK strain, were incubated (48 h) in inducing (galactose-containing) medium, and analyzed for cell morphology by DIC microscopy (left panel), and for TDP-43-GFP expression by epifluorescence (right panel). Shown images are representative of three biological replicates for each strain. Scale bar, 10 μm. **(B)** Cell morphology defects in the indicated strains were calculated by measuring the ratio between the cell major and minor axes (length/width ratio) from DIC micrographs. *n* = 6 (CENPK), 7 (TDP 1C), 8 (TDP 2C) (with each replicate representing the mean value from 60 to 80 cells), **p < 0.01, Kruskal–Wallis test followed by a Dunn’s *post hoc* test. **(C)** Viability assay in yeast strains carrying two copies of either WT or ALS-associated M337V or Q331K missense mutants of TDP-43. Yeast cells (OD_600_ = 1) of each strain were serially diluted (10-fold) and spotted onto either inducing medium plates (galactose, TDP-43 on), or repressing condition as control (glucose, TDP-43 off), and incubated at 30°C for 5 days. Data are representative of three experiments yielding comparable results. **(D)** The growth rate of the above strains (M337V and Q331K TDP 2C) was measured as described in the legend to Figure 1B. *n* = 9 for each strain, **p < 0.01, Kruskal–Wallis test followed by a Dunn’s *post hoc* test. **(E)** Yeast shape alteration by the expression (two genomic copies, 2C) of either M337V or Q331K TDP-43 mutants was determined by quantitative morphometric evaluation, as described in **(B)**. *n* = 8 for each strain, *p < 0.05, ***p < 0.001 Kruskal–Wallis test followed by a Dunn’s *post hoc* test.

It has been recently reported that the overexpression of human TDP-43 also affects cellular morphology in different yeast strains (i.e., BY4741 and W303), although the molecular mechanisms underlying such a defect remained unsolved (Park et al., 2017). We thus analyzed by optical microscopy the cell shape of yeast cells expressing TDP-43 from one or two integrated copies (after 48 h of induction in galactose), confirming that, in both cases, the expression of TDP-43 markedly perturbed cell morphology also in the CENPK genetic background, as already evident by visual inspection (Figure 2A). A quantification of cell ellipticity (roughly measured as the ratio between the long and the short cell axis) substantiated a significant shape defect of the TDP-43-expressing yeast strains (Figure 2B). Intriguingly, we did not observe such a morphologic phenotype in yeast cells expressing the ALS- related proteins FUS/TLS or C9orf72-derived PR dipeptide repeats (DPRs, PR50) (data not shown).

A strict correlation between the elongated phenotype and cell death, however, is ruled out by the observation that such morphological defect also appears in yeast cells expressing non-toxic levels of TDP-43 (i.e., TDP 1C, Figures 2A,B).

In addition to WT TDP-43, we also generated yeast strains carrying either one or two chromosomal copies of (GFP-tagged) human TDP-43 bearing the ALS-related Q331K and M337V missense mutations that are known to exacerbate TDP-43 cytotoxicity (Johnson et al., 2009). These mutants resulted in effects that were comparable to those produced by the same amount of the WT protein. Indeed, a single allelic copy of the transgene did not affect yeast viability but induced the elongated morphologic phenotype (Supplementary Figure 3), while two copies of both the TDP-43 mutant transgenes exerted detrimental effects on cell viability (Figure 2C), growth rate (Figure 2D), and cell morphology (Figure 2E).

The above data demonstrated that expression of WT or ALS-associated mutant human TDP-43 markedly affects cell viability and growth in yeast strains carrying two or more copies of the transgene, while the expression of TDP-43 (either WT or mutated) from a single genomic copy was not sufficient to cause cell death, pointing to a saturating effect of two transgene copies for TDP-43 cytotoxicity.

This experimental evidence validates the use of the here presented yeast strains as valuable cell models for the identification of biological factors and/or chemical compounds that can rescue TDP-43-dependent harm.

### TDP-43 Cytotoxicity Is Suppressed by Nucleolin (NCL)

Once established, we used such yeast models for TDP-43 proteinopathies to test the possible effects of NCL, which – as detailed in the Introduction – could be a promising player in the mechanisms of TDP-43 cytotoxicity. To this purpose, we overexpressed NCL (as a fusion construct with the red fluorescent protein mKate2) in the yeast strains bearing two or three integrated chromosomal copies of the TDP-43-GFP construct (WT or mutated), by means of an ectopic MC plasmid driven by a galactose-inducible promoter.

**FIGURE 3:**
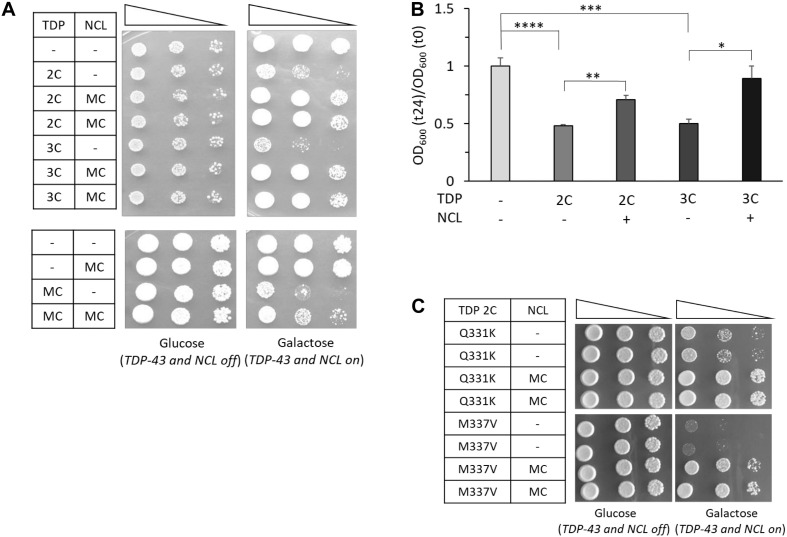
Overexpression of human NCL antagonizes the toxicity of WT and mutated TDP-43 in yeast cells. **(A)** Yeast CENPK cells carrying two (2C) or three (3C) genomic copies of TDP-43 transgene (upper panel) or containing TDP-43 multi-copy plasmid (MC) (lower panel), were transformed to co-express the human NCL protein by galactose-inducible vectors (MC). Yeast cells (OD_600_ = 1) of each strain were serially diluted (10-fold) and spotted onto either inducing medium plates (galactose, TDP-43/NCL on), or repressing condition as control (glucose, TDP-43/NCL off), and incubated at 30°C for 5 days (3 days for glucose plates of the upper panel). Data are representative of 3 experiments yielding comparable results. **(B)** The growth rate of the yeast strains of **(A)** was quantitatively determined as previously described (see Figure 1B). *n* = 9 for each strain. ^*⁣*⁣**^p < 0.0001, ^∗∗∗^*p* < 0.001, ^∗∗^*p* < 0.01, ^∗^*p* < 0.05, Kruskal–Wallis test followed by a Dunn’s *post hoc* test. **(C)** Viability assay in yeast strains carrying two genomic copies of mutant TDP-43 (M337V and Q331K) and transformed with NCL multi-copy plasmid (MC), or the empty vector (–) as control, performed as described above. Plates were incubated at 30°C for 5 days. Data are representative of three experiments yielding comparable results.

Both cell viability (Figure 3A) and growth rate (Figure 3B) assays demonstrated that NCL overexpression was able to rescue the negative effects of WT TDP-43-GFP overexpression from two, three or more (MC plasmid) gene copies. While NCL by itself did not result in any appreciable effect on cell viability (Figure 3A), the protein also prevented the lethality induced by two chromosomally integrated copies of the Q331K and M337V TDP-43 mutants (Figure 3C). Since we previously demonstrated that TDP-43 cytotoxicity strictly depends on the protein expression levels, we firstly ruled out the possibility that the rescue of the severe TDP-43-induced phenotypes by NCL could be caused by the downregulation of TDP-43-GFP amounts by NCL-mKate2 co-expression (Supplementary Figure 4).

Notably, we also observed that the genetic 1:1 stoichiometry of NCL with respect to TDP-43 was not sufficient to prevent TDP-43 cytotoxicity, as demonstrated by the loss of viability of cells carrying two integrated chromosomal copies of both TDP-43-GFP and NCL-mKate2 constructs, driven by the same galactose-inducible promoter (Supplementary Figure 5).

**FIGURE 4:**
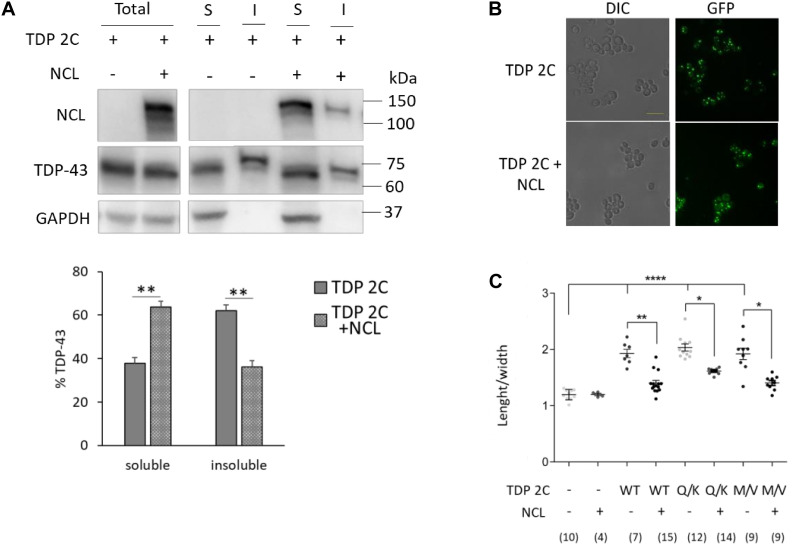
NCL overexpression reduced the detergent-insoluble TDP-43 fraction and reversed the cell shape alteration caused by TDP-43 in yeast cells. **(A)** Yeast cells carrying two copies of TDP-43 transgene (TDP 2C), transformed with either empty vector (–), or a MC plasmid encoding NCL (+), were incubated 6 h in galactose-inducing medium, and subjected to biochemical separation of RIPA-soluble and -insoluble protein fractions. Total, soluble (S), and insoluble (I) fractions were analyzed by WB for the presence of NCL, TDP-43, or GAPDH (upper panel). The lower panel reports the densitometric analysis of TDP-43-immunoreactive bands in RIPA-soluble and -insoluble fractions. Data are reported as mean ± SEM, *n* = 5, **p < 0.01, unpaired two-tailed Student’s *t*-test. Other details are as reported in the legend to Figure 1D. **(B)** The TDP 2C and the TDP 2C + NCL strains (as in **A**) were incubated 6 h in inducing medium, and analyzed for cell morphology by DIC microscopy (left panel), and for TDP-43-GFP expression by epifluorescence (right panel). Shown images are representative of three biological replicates for each strain. *Scale bar*, 10 μm. **(C)** Cell morphometry (as in the legend to Figure 2B) of yeast strains carrying two copies of either WT, or mutant (Q331K, M337V) TDP-43 transgene, transformed with either empty vector (–) or the MC plasmid coding for NCL (+). *p < 0.05, **p < 0.01, ****p < 0.0001, Kruskal–Wallis test followed by a Dunn’s *post hoc* test (n for each condition being indicated in the bottom part of the panel).

In the attempt to shed light on the ways by which NCL prevented TDP-43 toxicity, we next investigated the effects of NCL overexpression on TDP-43 behavior and the TDP-43-related morphologic phenotype in the TDP 2C strain. By biochemical fractionation assays, we found that the co-expression of NCL significantly increased the detergent-soluble fraction of WT TDP-43 (Figure 4A). Quite surprisingly, however, NCL did not apparently modified the cellular distribution pattern of TDP-43-GFP in multiple discrete foci observed by fluorescence microscopy (Figure 4B), suggesting that the detergent-insoluble TDP-43 assemblies differs from the species forming fluorescent punctae. We also observed that the overexpression of NCL substantially (albeit not fully) reverted the morphological alterations of yeast cells triggered by two chromosomal copies of either WT and mutant (Q331K and M337V) TDP-43, as indicated by the significant recovery of the normal cellular shape (Figure 4C).

Notably, untagged NCL, expressed by a MC plasmid, still retained the capability to counteract the toxicity of untagged TDP-43, as demonstrated by cell viability assays (Supplementary Figure 6), ruling out any potential effect of the GFP and mKate2 tags in the behavior of TDP-43 and NCL, respectively.

Collectively taken, the above data indicated that NCL overexpression was able to potently counteract the toxic effects of TDP-43 in yeast cells, and that such beneficial effect could be related to the aggregation dynamics of TDP-43, i.e., NCL reduced the detergent-insoluble fraction of TDP-43.

**FIGURE 5:**
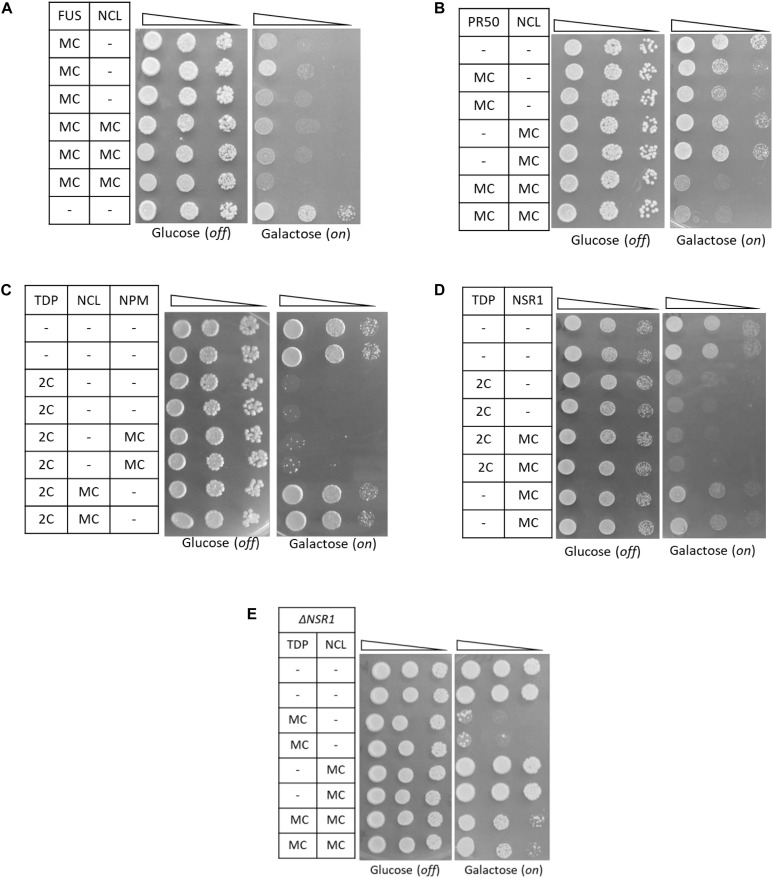
The functional relationship between TDP-43 and NCL in yeast is highly specific. **(A,B)** Yeast CENPK cells were co-transformed with a MC plasmid coding for FUS **(A)**, or the C9orf72-PR50 repeats **(B)**, together with the MC plasmid for NCL, or with empty vectors as negative controls (–). Yeast cells (OD_600_ = 1) of each strain were serially diluted (10-fold) and spotted onto either inducing medium plates (galactose, FUS/PR50/NCL on), or repressing condition as control (glucose, FUS/PR50/NCL off), and incubated at 30°C for 3 days. Data are representative of three experiments yielding comparable results. **(C,D)** Spotting assay [as in panels **(A,B)**] of yeast strains carrying two genomic copies of TDP-43 transgene (TDP 2C), or unmodified CENPK (–), both transformed with MC plasmids encoding either NCL, its functional partner nucleophosmin (NPM), its putative ortholog in yeast (NSR1) or the empty vector as control (–). **(E)** Spotting assay (as above) of the yeast CENPK strain carrying the deletion of the NSR1 gene (Δ*NSR1*), co-transformed with multi-copy plasmid (MC) overexpressing either TDP-43 (TDP), NCL, or a control empty vector (–). In all panels data are representative of three experiments yielding comparable results.

Additional experiments demonstrated that the relationship between TDP-43 and NCL was highly specific. Indeed, NCL overexpression failed to rescue the cytotoxicity caused in yeast cells by the expression of other ALS-related proteins (i.e., FUS/TLS and C9orf72 PR50 DPRs) by multicopy plasmids (Figures 5A,B, respectively). On the other hand, TDP-43 cytotoxicity (in the two-copy strain) was not alleviated by the overexpression of other human NCL-related nucleolar proteins, such as its functional partner nucleophosmin (NPM, Scott and Oeffinger, 2016), or the yeast NCL putative ortholog Nsr1p (Lee et al., 1991; Figures 5C,D, respectively). Since Nsr1p had been previously found to weakly enhance the toxicity of C9orf72-DPRs in yeast (Jovičič et al., 2015), we also explored TDP-43 toxicity (and the effects of NCL co-expression) in a mutant yeast strain bearing multiple TDP-43-GFP gene copies and depleted of the *NSR1* gene. We found that *NSR1* deletion did not impinge on the toxic phenotype induced by TDP-43 overexpression and that the ability of NCL to suppress TDP-43 toxicity was unperturbed in the Δ*NSR1* genetic background (Figure 5E).

### Mechanisms of the NCL Suppressive Effect on TDP-43 Toxicity

We then applied different approaches aimed at exploring the mechanisms by which NCL was able to counteract TDP-43-related toxic phenotypes.

#### Proteomic Analysis of TDP-43- and TDP-43/NCL-Expressing Yeast Cells

We firstly performed, by MS, a TMT-based proteomic profiling of the TDP 1C strain compared to the unmodified control strain (i.e., CENPK), both grown to log phase in raffinose medium (non-inducing) and transferred to galactose medium for 24 h to induce transgene expression. We chose to run the preliminary untargeted MS analysis on the TDP 1C strain, in which TDP-43 expression levels were sub-lethal, in order to possibly identify cell pathways induced by TDP-43 (and that NCL is able to counteract) whilst avoiding proteome alterations due to secondary events in downstream cell death pathways. By this assay, a total of 631 proteins were properly identified and quantified in all biological replicates of both strains. Interestingly, only 38 proteins were either significantly (*p*-value < 0.05) up-regulated (TDP-43/CENPK ratio > 1.33, 28 proteins) or down-regulated (TDP-43/CENPK ratio < 0.77, 10 proteins) in the TDP 1C strain (Supplementary Table 8).

**FIGURE 6:**
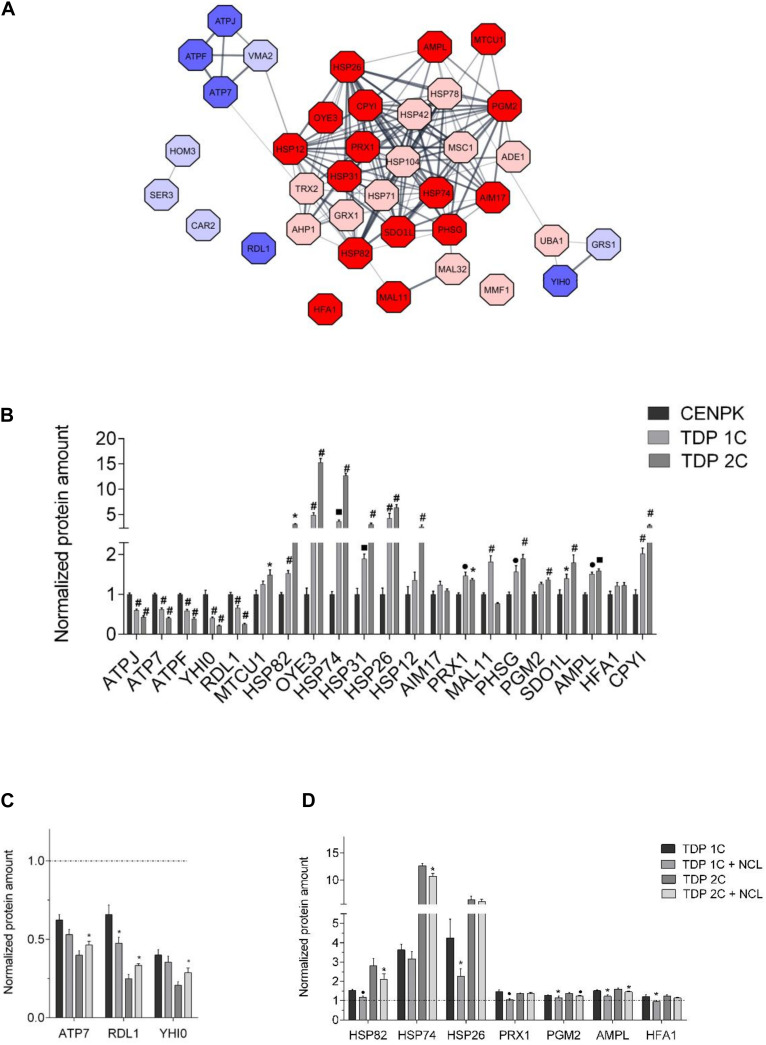
NCL rescues the expression of some proteins altered by TDP-43 in yeast cells. **(A)** Protein interaction network of the 38 proteins found to be differentially expressed (cut-off of TDP 1C/CENPK ratio > 1.33 or < 0.77) in the TDP 1C strain by the tandem mass tag MS/MS-based analysis. Up- and down-regulated proteins in TDP 1C are represented as red or blue nodes, respectively, with color intensity being representative of TDP/CENPK ratio value (i.e., dark red and blue colors indicate a TDP-43/CENPK ratio > 1.50 or <0.66, respectively, for a total of 21 proteins). The thickness of lines indicates the strength of association between protein nodes. **(B)** The relative abundance of the 21 proteins found to be mostly highly altered in the TDP 1C strain by the above untargeted proteomic approach was assessed by parallel reaction monitoring (PRM) assay in both TDP 1C and TDP 2C strains. Protein amounts are normalized to those measured in the control CENPK strain. *n* = 9; *p < 0.05, ^•^*p* < 0.01, ■ *p* < 0.001, #*p* < 0.0001, two-way ANOVA with Holm–Sidak correction for comparison of TDP 2C and TDP 1C to CENPK. **(C)** The effects of NCL-mKate2 expression on the abundance of down-regulated (left panel) or up-regulated (right panel) proteins in the TDP 1C and TDP 2C strains were assessed by PRM analysis. **(D)** Protein amounts are normalized to those measured in the CENPK strain (dashed line). *n* = 9; ^∗^*p* < 0.05, ^•^ *p* < 0.01 two-way ANOVA with Holm–Sidak correction for comparison of TDP 1C to TDP 1C + NCL and TDP 2C to TDP 2C + NCL.

In order to clarify which cellular processes could be altered by TDP-43 expression, we searched for interaction networks among the above 38 deregulated proteins in the TDP 1C strain through a computational analysis using the STRING software (Figure 6A). Such a functional enrichment analysis indicated that these deregulated proteins were strictly correlated (PPI enrichment *p*-value < 10^–16^) and that the vast majority of them belonged to discrete clusters. Notably, a gene ontology (GO) analysis showed that the most enriched cluster of up-regulated proteins included proteins involved in cellular response to stress, such as heat and oxidative stress, and oxidant detoxification (Supplementary Table 9).

We chose the 21 most up/down-regulated proteins (TDP-43/CENPK ratio > 1.50 and <0.66, respectively, Supplementary Table 8) for a subsequent targeted validation study by a MS-based parallel reaction monitoring (PRM) approach, in both TDP 1C and 2C strains compared with control cells. This analysis confirmed the deregulation found by the previous untargeted approach for one protein (MAL11) out of the chosen 21 in the 1C strain only, three proteins in the 2C strain only [MTCU1, HFA1, heat shock protein (HSP)12] and 15 proteins in both transgenic strains (Figure 6B). Again, most of up-regulated proteins were those implicated in stress response by the previous GO analysis, while most of the down-regulated proteins were involved in proton transmembrane transport and in biosynthetic processes (Supplementary Table 9). Conversely, the PRM analysis did not confirm the deregulation for two protein (AIM17; HFA1) in either transgenic strain (Figure 6B).

The PRM approach was also used to assess the effects of NCL co-expression (by a MC plasmid) on the levels of the subset of 19 proteins that were mostly deregulated by TDP-43 expression (in both the 1C and 2C strains) according to the unbiased proteomic analysis, and whose alteration was confirmed by the validation study of Figure 6B. We decided to exclude MAL11 and OYE3 from this analysis because MAL11 amounts were confirmed to be increased in the TDP 1C but not in the TDP 2C strain (Figure 6B), while OYE3 is a yeast NADPH oxidoreductase with no *bona fide* orthologs in mammals. Remarkably, we found that NCL partially restored the effects of TDP-43 on the expression of four proteins out of the chosen 19 in the 1C strain, five proteins in the 2C strain and one protein (HSP82, the yeast homolog of mammalian HSP90 protein) in both transgenic strains (Figure 6C) and that most of these proteins belonged to the stress response GO cluster (Supplementary Table 9).

In summary, the MS data indicated that the expression of TDP-43 (in the TDP 1C strain) triggered weak changes (∼6%) of the whole yeast proteome, resulting, however, in the significant up-regulation of specific proteins implicated in the response to oxidative and heat stress, and in protein folding and refolding processes that may be related to a proteostatic stress induced by TDP-43 expression. Most importantly, we also found that NCL brought the levels of some of these proteins in TDP-43-expressing strains toward the values observed in control cells, suggesting that it could decrease in some ways the proteostatic stress associated to TDP-43 intoxication.

#### NCL Cellular Localization

**FIGURE 7:**
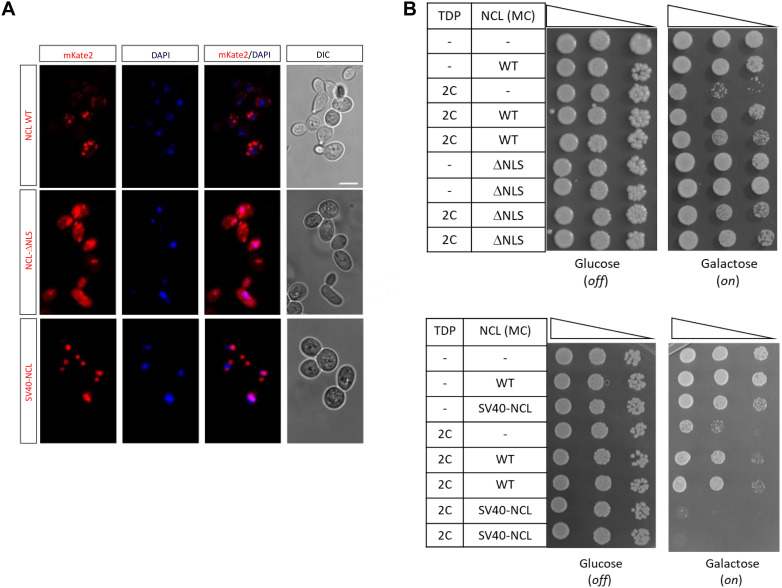
An extra-nuclear NCL fraction is probably responsible for the rescue of TDP-43 toxicity. **(A)** Yeast CENPK cells were transformed with the MC plasmids coding for the mKate2 red fluorescent protein fused to either NCL full-length protein (WT), lacking the nuclear localization signal (NCL-ΔNLS) or tagged with the SV40 target sequence (SV40-NCL). After 24 h incubation in inducing (galactose) medium, yeast cells were fixed, and analyzed by confocal microscopy to observe the NCL-mKate2 expression (left), the nuclear compartment (DAPI), and cell morphology (DIC, right). Images are representative of three biological replicates for each strain. Scale bar, 5 μm. **(B)** Yeast strains carrying two genomic copies of TDP-43 transgene (TDP 2C), or unmodified CENPK (–), were transformed with MC plasmids driving the overexpression of NCL, either WT, lacking the nuclear localization signal (ΔNLS, upper panel), or fused to the SV40 sequence (SV40-NCL, bottom panel). Yeast cells (OD_600_ = 1) of each strain were serially diluted (10-fold) and spotted onto either inducing medium plates (galactose, expression on), or repressing condition as control (glucose, expression off), and incubated at 30°C for 5 days. Data are representative of 3 experiments yielding comparable results.

As mentioned, human NCL prevalently localizes in the nucleolar compartment where it plays multiple roles (e.g., ribosome biogenesis), but it may also translocate outside the nucleus taking part to nucleocytoplasmic trafficking (Jia et al., 2017). We therefore addressed the issue of whether the NCL-mediated suppression of TDP-43 noxious effects required the nuclear/nucleolar localization of the protein, by removing the nuclear localization sequence (NLS) of NCL (amino acids 279–298). Confocal microscopy showed that NLS deletion profoundly modified NCL localization, by conferring to the protein a diffuse cell distribution pattern, while SV40 tagging resulted in a more evident nuclear localization (Figure 7A). Importantly, viability assays demonstrated that the NCL-ΔNLS mutant still retains the ability to rescue TDP-43 toxicity (Figure 7B, upper panel). Conversely, the forced retention of NCL within the nucleus, by tagging the protein with the additional SV40 NLS sequence, resulted in the complete loss of NCL antagonizing effects against TDP-43 (Figure 7B, lower panel). Such a finding was further supported by the observation that chimeric proteins obtained by fusing NCL to nuclear-resident protein (i.e., the activation domain of GAL4 transcription factor) was completely unable to rescue TDP-43 cytotoxicity (Supplementary Figure 7). These results strongly suggested the functional implication of the extranuclear fraction of NCL in suppressing TDP-43 toxicity in yeast cells.

#### TDP-43/NCL Physical Interaction

The above described genetic/functional interaction between TDP-43 and NCL might also be related to a physical interaction between the two proteins, considering that both proteins, although being mainly nuclear, may also localize in the cytosol. This possibility was suggested by a previous study showing – by immunoprecipitation of overexpressed TDP-43 followed by identification of co-purified proteins by MS – that NCL was a physical interactor of TDP-43 in HEK293T cells (Freibaum et al., 2010). We thus assayed if such a TDP-43-NCL physical association also occurred in yeast cells co-expressing TDP-43-GFP (in the strain bearing two chromosomally integrated copies of the transgene) and NCL-mKate2. By co-IP assays we found that a significant amount of the TDP-43-GFP chimera bound to NCL as demonstrated by its presence in the NCL-immunoprecipitated fraction (Supplementary Figure 8A). Consistently, proteins co-immunoprecipitated by an anti-GFP antibody (pulling down the TDP-43-GFP chimera) contained NCL-mKate2, and – vice versa – TDP-43-GFP co-immunoprecipitated with NCL-mKate2 by an anti-mKate2 antibody, providing further support to the interaction of the two proteins in yeast (Supplementary Figure 8B).

### NCL Antagonizes TDP-43 Cytotoxicity and Aggregation in Human Cells, Promoting TDP-43 Nuclear Localization

We next asked if NCL was able to suppress the TDP-43-induced phenotypes observed in the yeast models also in mammalian cells. To answer this question, we overexpressed human TDP-43 and NCL in the immortalized human cell line HEK293T by transient transfection with plasmids encoding fluorescence-tagged TDP-43-mKate2 and NCL-GFP chimeric constructs (see Supplementary Table 2 and Supplementary Methods).

**FIGURE 8:**
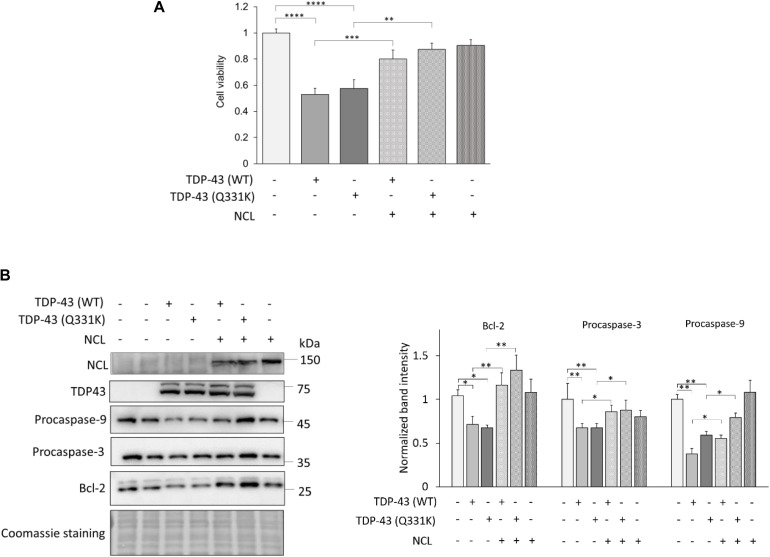
NCL rescues apoptosis induced by TDP-43 overexpression in human HEK293T cells. **(A)** Cell viability of HEK293T cells transiently expressing TDP-43-mKate2 [either WT or bearing the Q331K missense mutation, TDP-43 (WT) and TDP-43 (Q331K), respectively] and NCL-GFP (NCL) (+). As controls (–), cells were co-transfected with plasmids encoding mKate2 and/or GFP only. Data were normalized to the mean value of control samples (–/–/–). *n* = 15; ^∗∗^*p* < 0.01, ^∗∗∗^*p* < 0.001, ^*⁣*⁣**^p < 0.0001, Kruskal–Wallis test followed by a Dunn’s *post hoc* test. **(B)** WB analysis of lysates of HEK293T cells co-transfected as in **(A)** using antibodies to GFP (recognizing the NCL-GFP chimera), TDP-43, the anti-apoptotic factor Bcl-2, and procaspase-3 and -9. The left panel shows a representative WB out of four biological replicates (i.e., different cell transfections) also showing the Coomassie blue staining of the membrane; the right panel reports the densitometric analysis of immunoreactive bands for Bcl-2, procaspase-3 and procaspase-9 in the different cell samples normalized to the optical density of the corresponding Coomassie blue-stained lane. *n* = 4; ^∗^*p* < 0.05, ^∗∗^*p* < 0.01, one-way ANOVA followed by Sidak’s multiple comparison test.

We firstly assessed the viability of transfected cells by the MTS assay. Cells overexpressing the TDP-43 (either WT or bearing the Q331K missense mutation) chimera were significantly less viable (around 50%) compared to control cells (i.e., cells co-transfected with plasmids encoding GFP and mKate-2 only, Figure 8A), highlighting the cytotoxic potential of TDP-43 overexpression in human cells, as already reported (Lanznaster et al., 2019). While the NCL-GFP construct by itself did not affect cell viability, it strikingly antagonized the deadly effects of both WT and mutant TDP-43 in co-transfected cells (Figure 8A), indicating that NCL exerts a protective effect against TDP-43 toxicity also in mammalian cells.

In order to better clarify the mechanisms of TDP-43-induced toxicity, we analyzed the activation of the intrinsic apoptotic pathway that has been implicated in TDP-43 proteinopathies (Wang et al., 2015; Vogt et al., 2018). To this purpose, we examined by WB the amount of the anti-apoptotic protein Bcl-2, and of procaspase-3 and procaspase-9 whose cleaved products are executors of the apoptotic process. We found that (both WT and Q331K) TDP-43 overexpression reduced the amounts of Bcl-2 and of full-length (inactive) caspases, demonstrating the activation of the intrinsic apoptotic pathway, which – importantly – was remarkably reversed by the co-expression of NCL (Figure 8B).

**FIGURE 9:**
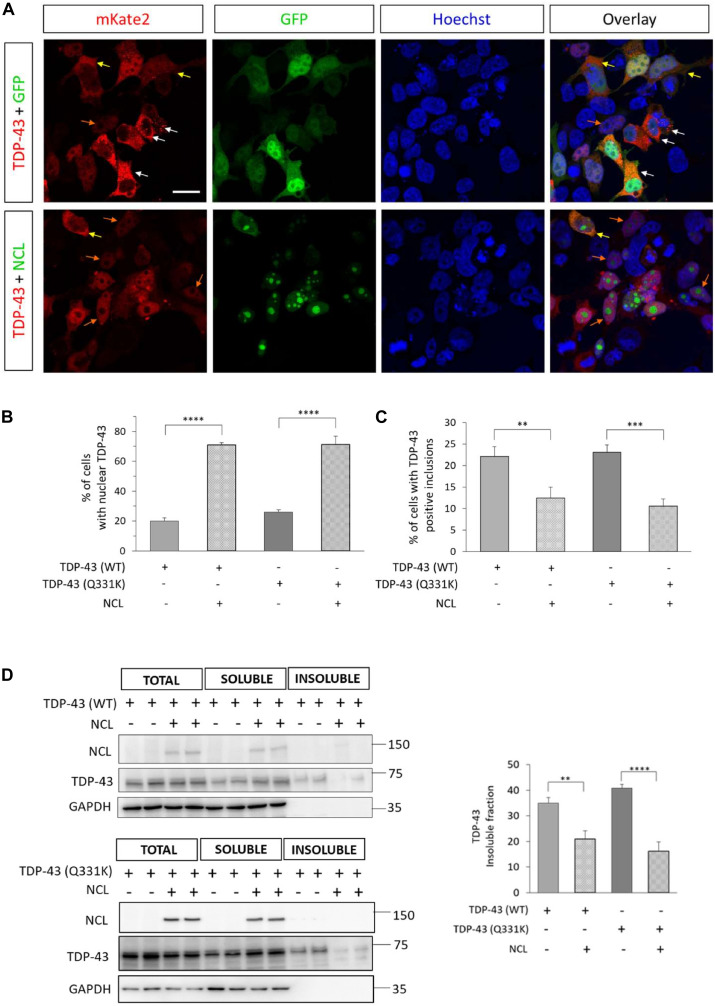
NCL rescued the nuclear localization of TDP-43, and reduced cytosolic inclusions and the detergent-insoluble fraction of TDP-43 in HEK293T cells. **(A)** Confocal microscopy analysis of HEK293T cells co-transfected with plasmids coding for human WT TDP-43 fused to mKate2 and either GFP alone (TDP-43 + GFP, upper panels), or the NCL-GFP chimera (TDP-43 + NCL, lower panels), and counterstained with the nuclear marker Hoechst. The reported micrographs, representative of three biological replicates, show cells with nuclear (orange arrows) or uniform cytosolic (yellow arrows) localization of TDP-43-mKate2, or intense discrete cytosolic red signals suggestive of the presence of TDP-43-mKate2 inclusions (white arrows). *Scale bar*, 20 μm. **(B)** Percentage of cells co-expressing TDP-43-mKate2 (either WT or bearing the Q331K mutation) and GFP (–) or NCL-GFP (+) having an exclusively nuclear mKate2 signal, with respect to the total of cells co-expressing both proteins. *n* = 3; ^*⁣*⁣**^p < 0.0001, one-way ANOVA followed by Sidak’s test for multiple comparisons. **(C)** Percentage of cells co-expressing either the WT or the Q331K mutant of TDP-43-mKate2 chimera and GFP (–) or NCL-GFP (+), presenting discrete (cytosolic) aggregation-like foci, with respect to the total of cells co-expressing both proteins. *n* = 3; ^∗∗^*p* < 0.01, ^∗∗∗^*p* < 0.001, one-way ANOVA followed by Sidak’s multiple comparison test. **(D)** Representative WBs (left panel) of RIPA-soluble and -insoluble fractions of lysates from HEK293T cells co-transfected with TDP-43-mKate2 (either WT, upper part, or carrying the Q331K mutation, lower part) and either GFP alone (±), or the NCL-GFP chimera (+/+). As control, total lysates were loaded. The presence of NCL-GFP (with anti-GFP antibody), TDP-43-mKate2 (with anti-TDP-43 antibody) and GAPDH (as control for detergent-soluble proteins) were analyzed. The percentage of insoluble TDP-43-mKate2 in the different analyzed samples is shown in the right panel. *n* = 5 for TDP-43(WT), *n* = 3 for TDP-43(Q331K); ^∗∗^*p* < 0.01, ^*⁣*⁣**^p < 0.0001, one-way ANOVA followed by Sidak’s multiple comparison test.

Since TDP-43 delocalization to the cytoplasm, and its consequent depletion from the nucleus, is a prominent pathological feature in ALS and related TDP-43 proteinopathies (Prasad et al., 2019), we then evaluated by confocal microscopy the cell distribution of overexpressed (WT or Q331K) TDP-43-mKate2 in HEK293T cells, in the absence or the presence of overexpressed NCL-GFP. As shown in Figures 9A,B, NCL overexpression remarkably increased the percentage of cells in which TDP-43 was correctly localized to the nucleus (from ∼20 to 25% in TDP-43-mKate2/GFP co-transfected cells to ∼70% in TDP-43-mKate2/NCL-GFP co-transfected cells for both WT and Q331K TDP-43), thereby reducing its (toxic) accumulation in the cytoplasm. In keeping with the idea that extra-nuclear delocalization of TDP-43 correlates with its accumulation in cytosolic inclusions, we found that the fraction of cells with (WT or mutant) TDP-43-mKate2 fluorescent foci was significantly lower (by ∼50%) in the presence of NCL-GFP (Figures 9A,C). The quantification of the detergent-soluble and -insoluble protein fractions of TDP-43-mKate2 in the absence or the presence of NCL-GFP by biochemical fractionation of HEK293T lysates was also consistent with this finding. Indeed, mirroring what observed in yeast cells, NCL was able to significantly reduce (from ∼35% to ∼20%) the insoluble TDP-43 fraction of both the WT and the Q331K TDP-43 chimeras (Figure 9D).

Taken together, the above data demonstrated that the overexpression of TDP-43 (either WT or bearing the ALS-associated Q331K missense mutation) was toxic to, and formed detergent-insoluble aggregates in, HEK293T cells and that NCL was able to attenuate both phenotypes, facilitating the nuclear localization of TDP-43. These data substantiated a functional role of NCL in antagonizing TDP-43 proteinopathies.

The finding that extranuclear NCL is prominent in abolishing TDP-43 toxicity in yeast (Figure 7), prompted us to apply to human cells the same genetic approach to force NCL localization either outside (i.e., NLS deletion) or inside (i.e., fusion to the SV40 nuclear targeting sequence) the nucleus. According to yeast data, we found that the NCL-ΔNLS mutant was still able to rescue the viability of TDP-43-expressing HEK293T cells, whereas TDP-43 toxicity was not significantly suppressed by the nuclear SV40-NCL chimeric protein (Supplementary Figure 9).

**FIGURE 10:**
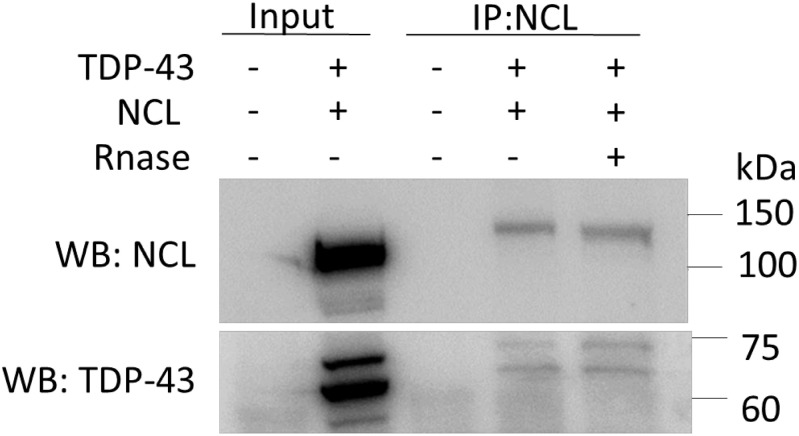
TDP-43-mKate2 and NCL-GFP co-immunoprecipitate in HEK293T cells independently from the presence of RNA. Proteins extracted from cells co-transfected with TDP-43-mKate2 and NCL-GFP expression vectors (+/+), or from non-transfected cells (–/–), were treated (+) or not (–) with RNase A and immunoprecipitated using the anti-NCL antibody. The immunocomplexes were analyzed by WB using either anti-GFP (upper panel) or anti-TDP-43 (lower panel) antibodies, showing the presence of both NCL-GFP (band between 100 and 150 kDa) and TDP-43-mKate2 (bands between 60 and 75 kDa) irrespective of RNase treatment. 20 μg of the total cell lysates (Input) were loaded as control. Shown results are representative of three different experiments.

Finally, following the co-IP observations in yeast cells (Supplementary Figure 8) and the previous finding that TDP-43 and NCL can interact in HEK293T cells (Freibaum et al., 2010), we checked if the physical association between the two proteins (i.e., TDP-43-mKate2 and NCL-GFP) also occurred in our experimental paradigm. Although we did never observe a clear colocalization of NCL and TDP-43 by fluorescence/confocal microscopy (e.g., see Figure 9), possibly due to the strong nucleolar NCL signal, the co-IP experiments reported in Figure 10 confirmed the interaction between the two proteins. This brought further support to the notion that the beneficial effects of NCL could be related to its physical association to TDP-43. Importantly, RNA – which binds both TDP-43 and NCL – does not seem to be involved in such an interaction, since RNase treatment of cell lysates did not hamper the co-IP of the two proteins.

## Discussion

TDP-43-mediated neurodegeneration is characterized by the depletion of nuclear TDP-43 and the concomitant accumulation of the protein in aberrant cytoplasmic assemblies. These two hallmarks suggested that TDP-43 proteinopathies arise from either a loss of function, by impairing the normal function of nuclear TDP-43 in regulating transcription and mRNA processing, or a gain of toxicity of the cytosolic fraction of the protein, or both (Lee et al., 2012; Cascella et al., 2016). Whatever the pathogenic relevance of TDP-43 aggregation, identifying modifiers of TDP-43 toxicity and understanding the cell pathways altered by TDP-43 cytosolic mislocalization and its accumulation into protein condensates could unveil novel therapeutic strategies for TDP43-related proteinopathies, such as ALS and FTD.

Here we showed that NCL, sharing different structural and functional attributes with TDP-43, is able to revert cell death and other phenotypes related to TDP-43 overexpression in both yeast and human *in vitro* cell models.

The use of the *S. cerevisiae* yeast model already provided important hints into NDs caused by protein misfolding and aggregation, including TDP-43 proteinopathies (Kryndushkin and Shewmaker, 2011). In this work, taking advantage of the CRISPR/Cas9 technique, we established a novel yeast model of TDP-43 proteinopathies that correlates TDP-43 toxicity with the number of transgene copies carried by yeast cells. Indeed, we demonstrated that two genomic copies of the human WT TDP-43 transgene were necessary and sufficient to cause complete lethality in yeast cells. Importantly, we correlated TDP-43-dependent cytotoxicity to the detergent solubility of the overexpressed protein, pinpointing the relevant role of TDP-43 aggregates/condensates for its toxic effects to yeast cells. Indeed, the majority of TDP-43 protein in cells carrying two or more transgene copies partitioned into the detergent-insoluble fraction, differently from fully viable yeast cells carrying one TDP-43 transgene copy.

We also analyzed the effects of two ALS-linked TDP-43 missense mutations (i.e., Q331K and M337V), that are known to increase TDP-43 toxicity in yeast cells (Johnson et al., 2009). However, according to a more recent study (Bolognesi et al., 2019), we found that both disease-associated mutants behaved like the WT protein, as no cytotoxicity was observed when mutant proteins were expressed by a single genomic copy. In addition, their effects to yeast viability in strains carrying two transgene copies were comparable to those of WT TDP-43, further supporting that TDP-43 cytotoxicity in yeast cells was dose-dependent (Armakola et al., 2011) and demonstrating a saturating effect of two copies of either WT or mutant TDP-43 transgene in the yeast genome.

Our models also recapitulated recently described alterations of yeast cell morphology caused by TDP-43 expression (Park et al., 2017), but the mechanisms and the significance of such a peculiar shape modification remain to be determined. With this respect it is important to highlight that under different stress conditions (e.g., nitrogen starvation) yeast cells can activate several signaling pathways to preserve cell survival converging to the transcriptional induction of specific genes, which may promote morphological alterations, such as filamentous growth (Cullen and Sprague, 2012). In addition, yeast cell shape can also be perturbed by dysfunctions of key cellular processes, such as autophagy (Song and Kumar, 2012) or nucleocytoplasmic trafficking (Künzler et al., 2001), both of which have already been associated to TDP-43 function/toxicity (Budini et al., 2017; Chou et al., 2018). Notably, such a morphologic phenotype was specifically determined by the presence of TDP-43, but not by other ALS-related proteins. Indeed, FUS/TLS or the C9orf72-derived PR dipeptide repeats (DPRs, PR50) – which have been previously reported to cause cytoplasmic inclusion foci and cell death in yeast (Fushimi et al., 2011; Jovičič et al., 2015; Chai and Gitler, 2018) – did not produce in our yeast models the elongated phenotype observed in TDP-43-expressing cells. Whatever the mechanism, the occurrence of such a morphologic phenotype also in yeast strains carrying a (non-toxic) single copy of the TDP-43 transgene suggested that it was unrelated to the lethal TDP-43 phenotype.

Taken together, the above findings demonstrated that the novel models here generated were able to recapitulate in a more stable genetic context the major effects of TDP-43 expression in yeast cells, i.e., cytotoxicity, increased propensity to protein aggregation/condensation, and specific morphological alterations, and were therefore suited for the identification of possible antagonists of TDP-43-induced cellular damage and to investigate the cell pathways thereof.

In this work, we focused our attention on NCL as a possible modifier of TDP-43 cytotoxicity. Indeed, NCL was reported as (one of) the most abundant nucleolar protein and is the first identified RNA binding protein able to shuttle from nucleus to cytoplasm, being involved in the transport of RNA molecules and proteins across the nuclear membrane (Jia et al., 2017). Importantly, NCL was also identified as a modifier of C9orf72-mediated cell toxicity in a *Drosophila* model (Lee et al., 2016), although no further investigation on this issue has been reported. Furthermore, NCL was included among the numerous TDP-43 interacting proteins involved in RNA metabolism which were identified through a global proteomic approach on HEK293T cells overexpressing TDP-43 (Freibaum et al., 2010).

Another link between NCL and ALS/FTD was suggested by the co-segregation of NCL and some RNAs (e.g., C9orf72) or RNA binding proteins (including TDP-43 and FUS/TLS), in RNA foci accumulated in ALS patient tissues, resulting in the altered expression of specific RNA targets, RNA misprocessing events and disruption of nucleolar functions (Haeusler et al., 2014; Zhou et al., 2014; Butti and Patten, 2018).

Moreover, NCL was also reported to play a role in the long-term maintenance of mature neurons (Hetman and Pietrzak, 2012), and has been related to other NDs (e.g., see Caudle et al., 2009).

Importantly, we confirmed in mammalian cells the evidence that NCL can potently counteract TDP-43 (WT or bearing ALS-related missense mutations) cytotoxicity and TDP-43 detergent-insolubility observed in our yeast models. Indeed, data from human HEK293T cells indicated that NCL overexpression markedly decreased the number of cells with cytoplasmic TDP-43 aggregates, while concurrently increasing the number of cells displaying proper TDP-43 nuclear localization. We further observed that the TDP-43-dependent activation of apoptotic cell death in human cells was significantly reduced by NCL co-expression, suggesting that NCL could suppress TDP-43 toxicity also by directly acting as an antiapoptotic factor, in light of its reported ability to stabilize the mRNA of the Bcl-2 anti-apoptotic factor (Ishimaru et al., 2010), and to modulate p53 signaling at multiple levels (Takagi et al., 2005; Bhatt et al., 2012). We indeed observed that NCL overexpression was able to rescue the normal Bcl-2 levels in TDP-43 overexpressing HEK293T cells, without, however, affecting Bcl-2 expression in cells expressing endogenous TDP-43 amounts.

However, in yeast cells, NCL was not able to counteract the cytotoxicity induced by the overexpression of FUS and C9orf72 DPR, both of which were recognized – similarly to TDP-43 – to induce cell death via the mitochondrial apoptotic pathway (Braun, 2012), suggesting a specific effect of NCL over TDP-43-mediated pathways. Thus, present and previous findings strongly suggest that NCL acts upstream of the apoptotic cascade triggered by TDP-43 overexpression, possibly by reducing the accumulation of cytosolic TDP-43 aggregates. In light of the reported activation of ER stress-related apoptotic cell death by TDP-43 (Vaccaro et al., 2013; Wang et al., 2015), one may also speculate that NCL interferes with the TDP-43/ER stress correlation.

Further studies are needed to establish the exact mechanisms by which NCL specifically target TDP-43 pathology. However, the observation that (both in yeast and mammalian cells) the removal of the NLS did not influence NCL ability to rescue TDP-43 toxicity, which was conversely abrogated by introducing an additional NLS signal forcing NCL retention in the nuclear compartment, strongly suggested that the extranuclear localization of NCL, possibly linked to its nucleocytoplasmic shuttling role, is required to suppress TDP-43 proteotoxicity.

According to the evidence provided by this work, a possible scenario could be proposed, whereby the extranuclear fraction of NCL interacts with TDP-43 in the cytosol, restoring its correct movement across the nuclear membrane and promoting its nuclear localization and solubility, therefore preventing the formation of cytosolic TDP-43 aggregates, and finally alleviating the proteostatic stress induced by TDP-43 overexpression and/or disease-related mutations.

An emerging theme in TDP-43 proteinopathies is the ability of the protein to undergo phase transitions, forming liquid-like condensates that may be more harmful to neurons than insoluble aggregates. The nature (e.g., liquid like vs. “solid” inclusions) and the cell localization (e.g., nuclear or cytoplasmic) of TDP-43-containing structures that NCL is able to cure may thus worth further investigation.

Concurrently, another sensible hypothesis entails that NCL exerts a chaperone-like activity for TDP-43, as already suggested for histones, whereby NCL helps histone nuclear import and storage when not assembled with DNA (Gaume et al., 2011). By this activity, NCL would promote TDP-43 proper folding and nuclear re-localization, thus preventing its cytosolic aggregation.

This hypothesis is supported by the MS analysis performed in yeast cells providing evidence that among numerous altered proteins by TDP-43 expression, the presence of NCL restored the normal levels of some HSPs. Interestingly, NCL restored the levels of HSP70 and of HSP82 (the yeast homolog of HSP90), both previously suggested to functionally interact with TDP-43 (Lackie et al., 2017). Possibly, this may be determined by NCL activity in transcription/translation processes or a compensatory NCL role as a molecular chaperone.

The targeted proteomic approach was based on a preliminary untargeted large-scale MS analysis of yeast cells, which identified several proteins dysregulated by TDP-43 expression. Although we may possibly have identified a higher number of dysregulated proteins in the toxic TDP 2C strain, we chose to run such a first large-scale study in the non-toxic TDP 1C strain in order to avoid foreseeable secondary whole proteome alterations related to cell death pathway activation. Such a choice was corroborated by the subsequent PRM targeted analysis, demonstrating that most of proteins involved in cell stress response, protein folding, and proton transport that we found to be modified in TDP 1C cells were equally altered in the (toxic) TDP 2C strain.

Specifically, we found in both strains the upregulated expression of proteins involved in the response to oxidative stress or also of some chaperone proteins belonging to the HSP subfamily involved in protein folding and stress granule disassembly, which may speculatively be associated to a compensative response to TDP-43-induced protein misfolding stress. Conversely, among the MS identified proteome no evident alteration was observed for proteins directly involved in nucleocytoplasmic trafficking, while – unfortunately – our analysis failed in detecting any protein possibly implicated in the filamentous yeast growth or autophagy. Importantly, however, we also found that TDP-43 expression in yeast is associated to a down-regulation of several subunits of ATP synthase, supporting the idea that TDP-43-induced damages may also involve mitochondrial disfunctions, as reported previously (Braun et al., 2011).

## Conclusion

Here, reported findings clearly identified NCL as a powerful modifier of TDP-43 proteotoxicity in both yeast and mammalian cells. Although studies are needed to assess if the above proposition still stands in ALS selectively vulnerable MNs and in *in vivo* ALS experimental settings, NCL seems a promising means to understand the mechanisms of TDP-43-associated proteinopathies and a possible therapeutic target for ALS and related disorders.

## Data Availability Statement

The mass spectrometry proteomics data have been deposited to the ProteomeXchange Consortium via the PRIDE partner repository with the dataset identifier PXD022432.

## Author Contributions

RL, AB, and CP: conceptualization. RL, RS, and CP: methodology. CP, RS, and RB: formal analysis. CP, RB, RS, MM, JA, and AM: investigation. FT, GS, and MM: resources. RL, AB, and CP: writing – original draft. RL, AB, CP, FT, MM, GS, and RS: writing – review and editing. RL and AB: supervision and funding acquisition. All authors contributed to the article and approved the submitted version.

## Conflict of Interest

The authors declare that the research was conducted in the absence of any commercial or financial relationships that could be construed as a potential conflict of interest.

## Abbreviations

ACN: acetonitrile
AD: Alzheimer’s disease
AGC: automatic gain control
ALS: amyotrophic lateral sclerosis
AUC: area under the curve
DPRs: dipeptide repeats
DTT: dithiothreitol
FTD: frontotemporal dementia
FUS/TLS: fused in sarcoma/translocated in liposarcoma
HCD: higher energy C-trap dissociation
HD: Hungtington’s disease
HPLC-HRMS/MS: high-performance liquid chromatography-high resolution tandem mass spectrometry
MC: multi-copy
NCL: nucleolin
NLS: nuclear localization signal
NDs: neurodegenerative diseases
NPM: nucleophosmin
PD: Parkinson’s disease
PRM: parallel reaction monitoring
RRM: RNA recognition motifs
TDP-43: transactive response DNA binding 43 kDa protein
TEAB: triethylammonium bicarbonate
TMT: tandem mass tag.
