# Supplementary material for: Nucleolin Rescues TDP-43 Toxicity in Yeast and Human Cell Models
Source: Front Cell Neurosci. 2021 Apr 12;15:625665. doi: 10.3389/fncel.2021.625665 (PMC8072491; doi:10.3389/fncel.2021.625665)
Supplement: Supplementary file 1 [file Data_Sheet_1.PDF]

Supplementary Material

Supplementary Figures

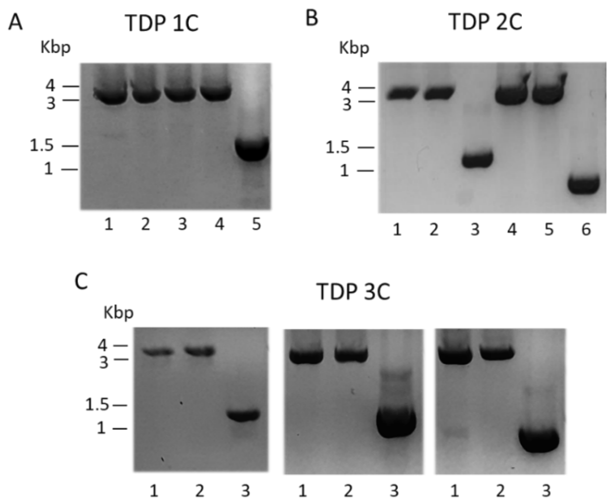

Supplementary Figure S1.

PCR analyses to verify the integration of 1, 2 or 3 copies of the P_{Gal1}-TDP-43-GFP-CYC1 TT cassette by CRISPR/Cas9 editing in the CEN.PK IMX672 yeast strain.

A. Agarose gel electrophoresis showing PCR amplifications of the genomic DNA extracted from four different TDP 1C colonies (lanes 1-4) obtained by the *in vivo* recombination of the P_{Gal1}-TDP-43-GFP-CYC1 TT (TDP) cassette in the *HIS3* locus (chromosome (chr) XV) of yeast cells. The unmodified CEN.PK IMX672 (hereafter abbreviated as CENPK) strain was used as control (lane 5). For PCR amplification, dgHIS3 forward (F) and reverse (R) primers (Supplementary Table 5) were designed outside the *HIS3* locus (-285 bp and + 485 bp, respectively) to differentiate between successful and non-successful integrations via PCR. The expected product size of the amplified PCR fragments is ~3500 bp in TDP 1C transformants and ~1500 bp in the non-edited strain.
B. PCR results of two selected TDP 2C colonies (lanes 1,2,4,5) and one unmodified CENPK strain (lanes 3,6*)* with dgHIS3 F and R primers (lanes 1-3) and with dgTRP1 F and R primers (lanes 4-6). To confirm the successful insertion of TDP cassette in *TRP1* locus (chr IV) of TDP 1C strain, dgTrp1 F and R primers (Supplementary Table 5) were designed outside of *TRP1* locus (-37, +270 bp, respectively). The expected product sizes of the amplified PCR fragments are ~3000 bp in TDP 2C transformants or ~1000 bp in CENPK strain. PCR validation with dgHIS3 F and R primers (lanes 1-3) was performed to confirm the presence of TDP cassette in the *HIS3* locus of TDP 2C colonies.
C. PCR products of two isolated colonies of TDP 3C strain (lanes 1,2 of each panel) and one unmodified CENPK strain (lanes 3 of each panel) with dgHis3 F and R (first panel), dgLeu2 F

and R (second panel) and dgTrp1 F and R (third panel) primers (Supplementary Table 5). TDP 3C was generated by Cas9-mediated integration of TDP cassette also in the *LEU2* locus (chr III) of TDP 2C strain. dgLeu2 F and R primers were designed outside of the *LEU2* locus (- 230 bp and + 245 bp) with an expected product sizes of the amplified PCR fragments of ~2900 bp in TDP 3C transformants or ~850 bp in the absence of any editing. PCR validation with dgHIS3 and dgTrp1 primers were performed to confirm the presence of TDP cassette in the *HIS3* and *TRP1* loci of TDP 2C colonies.

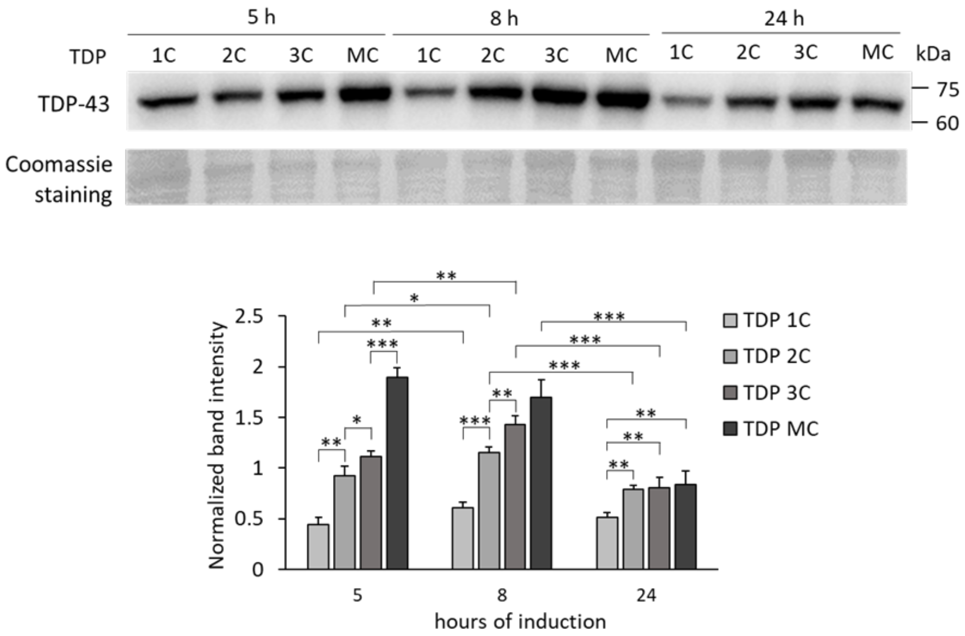

Supplementary Figure S2.

Evaluation of TDP-43-GFP expression in the different yeast strains generated by genome editing or transformation with an ectopic plasmid.

After 5, 8, and 24h of TDP-43-GFP expression induction by different yeast strains (TDP 1C, 2C, 3C, or expressing TDP-43-GFP by a non-integrative multi-copy plasmid (TPD MC)), protein lysates were analysed by Western blot (WB) using an anti-TDP-43 antibody. The upper panel shows a representative WB and the related Coomassie blue staining, while the lower panel reports the densitometric analysis of TDP-43-GFP immunoreactive bands. Data are mean ± SEM. n=5 for each strain, *p<0.05, **p<0.01, ***p<0.001 paired, two-tailed Student's t-test.

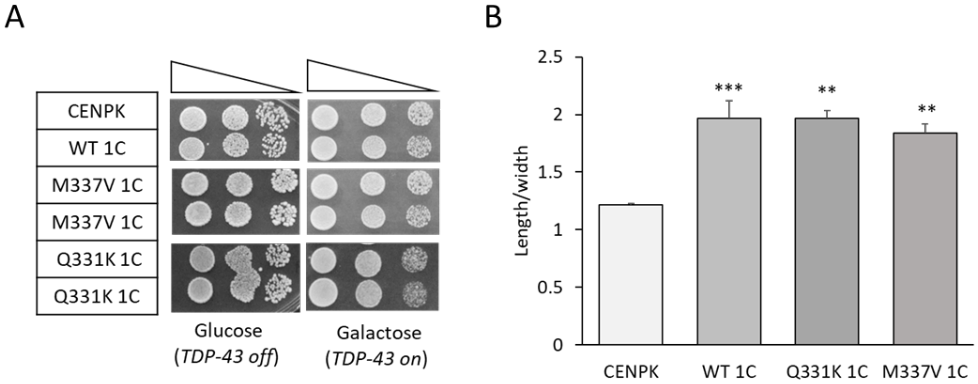

Supplementary Figure S3.

Yeast cells carrying 1 copy of the genome-integrated ALS-related TDP-43 mutations behave as the WT TDP-43 1C transgene.

A. Viability assay of yeast strains carrying in the genome one copy (1C) of either wild-type (WT) or ALS-related TDP-43 mutations (M337V or Q331K). Yeast cells (OD_{600}=1) of each strain were serially diluted (10-fold) and spotted onto either inducing medium plates (galactose, TDP-43 on), or repressing condition as control (glucose, TDP-43 off), and incubated at 30 °C for 3 days. Unmodified (CENPK) strain was used as positive control for cell growth. Shown images are representative of 3 different experiments.

B. Cell morphology defects of the strains of the Fig S3A were calculated by measuring the ratio between the cell major and minor axes (ellipticity) from DIC micrographs. Data are mean ± SEM, n=6 biological replicates (each biological replicate value represents the mean of the length-to-width ratio of ~50 cells); **p<0.01, ***p<0.001 Kruskal-Wallis test followed by a Dunn's post hoc test.

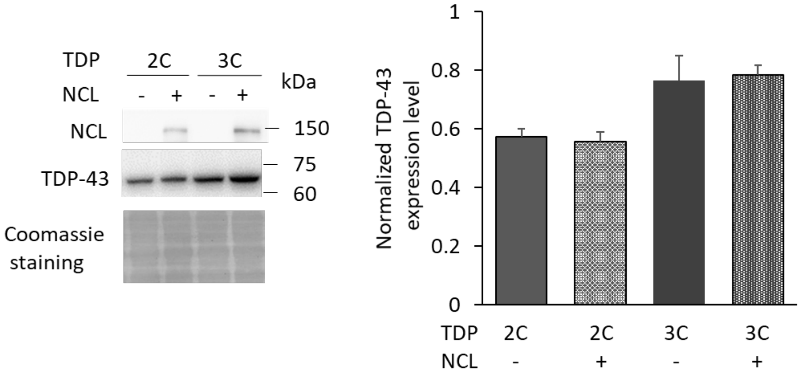

Supplementary Figure S4.

NCL-mKate2 expression does not alter the amount of TDP-43-GFP in TDP 2C and 3C yeast strains.

TDP 2C and 3C yeast strains transformed with a non-integrative multi-copy plasmid coding for NCL-mKate2 (+), or the empty vector (-), were grown in a galactose-rich medium (24 h) to induce transgene expression. Cell lysates were analysed by WB with an antibody to TDP-43 or NCL. In the left panel a representative immunoblot is reported, showing NCL-mKate2 and TDP-43-GFP immunoreactive bands, and the corresponding Coomassie-stained lanes, in the different samples, as indicated. The right panel, reporting the densitometric analysis of TDP-43-GFP. Data are mean ± SEM. n=5 for each strain.

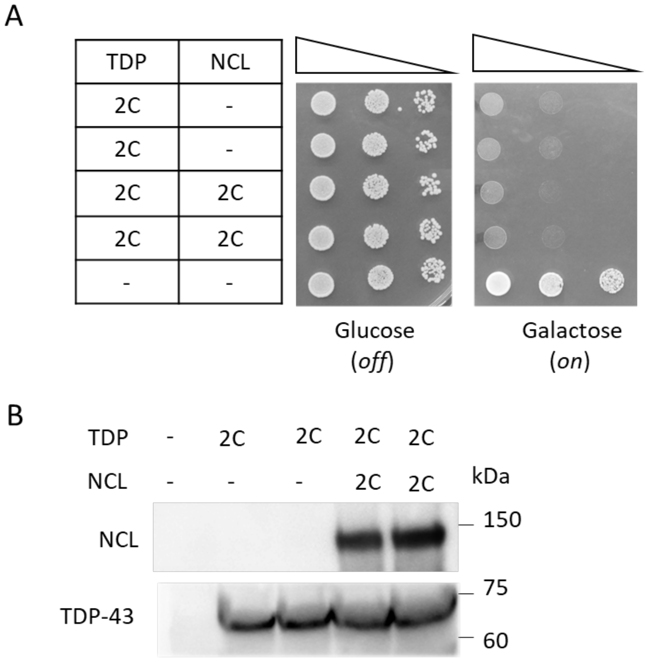

Supplementary Figure S5.

Two genome-integrated copies of the NCL-mKate2 transgene are not sufficient to protect from TDP-43 toxicity in the TDP 2C yeast strain.

A. Viability assay of yeast strains carrying in the genome two copies (2C) of both TDP-43 and NCL human transgenes. Yeast cells (OD_{600}=1) of each strain were serially diluted (10-fold) and spotted onto either inducing medium plates (galactose, TDP-43/NCL on), or repressing condition as control (glucose, TDP-43/NCL off), and incubated at 30 °C for 3 days. Unmodified (CENPK) strain was used as positive control for cell growth. Shown images are representative of 3 different experiments.
B. The yeast strains of Fig S5A were incubated in galactose inducing medium for 24 h, and total protein lysates were subjected to Western Blot analysis using antibodies anti-NCL (upper panel) and anti-TDP-43 (bottom panel). Unmodified CENPK strain (-/-) was considered as negative control.

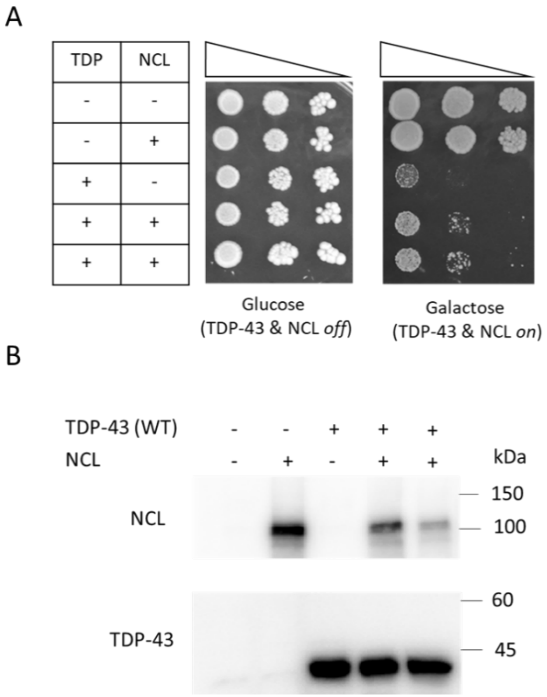

Supplementary Figure S6

The expression of native NCL is still able to counteract TDP-43 induced cell death in yeast.

A. Viability assay of yeast CENPK cells co-transformed with multi-copy, galactose-inducible plasmids overexpressing either native TDP-43 and NCL proteins (i.e., untagged) (+), or the empty vectors (-). Yeast cells (OD_{600}=1) of each strain were serially diluted (10-fold) and spotted onto either inducing medium plates (galactose, TDP-43/NCL on), or repressing condition as control (glucose, TDP-43/NCL off), and incubated at 30 °C for 3 days. Shown images are representative of 3 different experiments.
B. WB analysis using anti-NCL and anti-TDP-43 antibodies to confirm the expression of native NCL and TDP-43 in transformed yeast cells analysed in panel A.

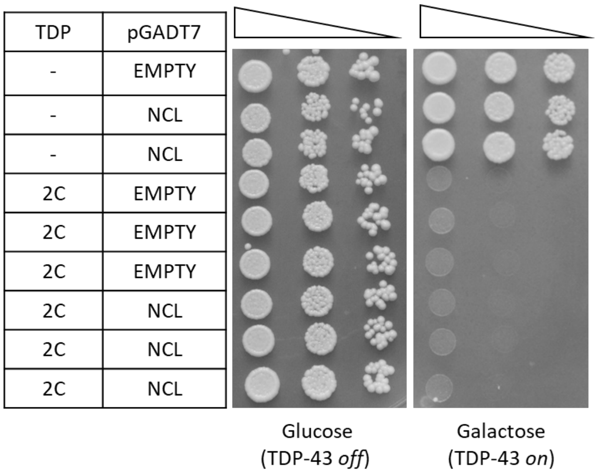

Supplementary Figure S7.

The forced retention of NCL into the nucleus abrogates NCL protective effects on TDP-43 toxicity.

Viability assay of yeast strains carrying in the genome two copies of human TDP-43 transgene (2C), transformed with either NCL-pGADT7 plasmid (NCL), expressing the Gal4(AD)-NCL chimeric protein, or the vector alone (EMPTY). Yeast cells (OD_{600}=1) of each strain were serially diluted (10-fold) and spotted onto either inducing medium plates (galactose, TDP-43 on), or repressing condition as control (glucose, TDP-43 off), and incubated at 30 °C for 3 days. Shown images are representative of 3 different experiments.

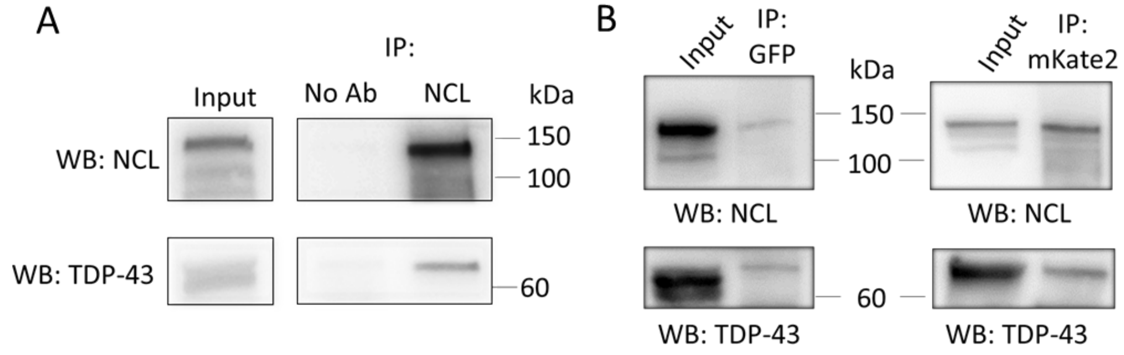

Supplementary Figure S8.

TDP-43 and NCL interact in an *in vitro* co-immunoprecipitation assay in yeast cells.

A. Cell lysates from the TDP 2C strain overexpressing the NCL-mKate2 chimera by a MC plasmid (collected after 8 h of culture in galactose, promoting transgene expression) were subjected to immunoprecipitation using an anti-NCL antibody. The immunoprecipitated fraction was then subjected to WB analysis using either anti-NCL (upper panels) or anti-TDP-43 (lower panels) antibodies, showing the presence of both NCL-mKate2 (band between 100 and 150 kDa) and TDP-43-GFP (band above 60 kDa). As controls, also a fraction of the total lysate (Input) and a precipitated fraction in the absence of the anti-NCL antibody (No Ab) were analysed. Shown data are representative of 3 different experiments.
B. Yeast cell lysates as in panel A were subjected to immunoprecipitation using either an anti-GFP (TDP-43-GFP pull-down, left panels) or anti-mKate2 (NCL-mKate2 pull-down, right panels) antibody, and immunoprecipitated fractions were subjected to WB analysis using anti-NCL (upper panels) or anti-TDP-43 (lower panels) antibodies. The presence of both NCL-mKate2 and TDP-43-GFP in both immunoprecipitated fractions provides further support to the physical interaction between the two chimeric proteins in yeast cells. In both approaches, a total lysate fraction (Input) was analysed as control.

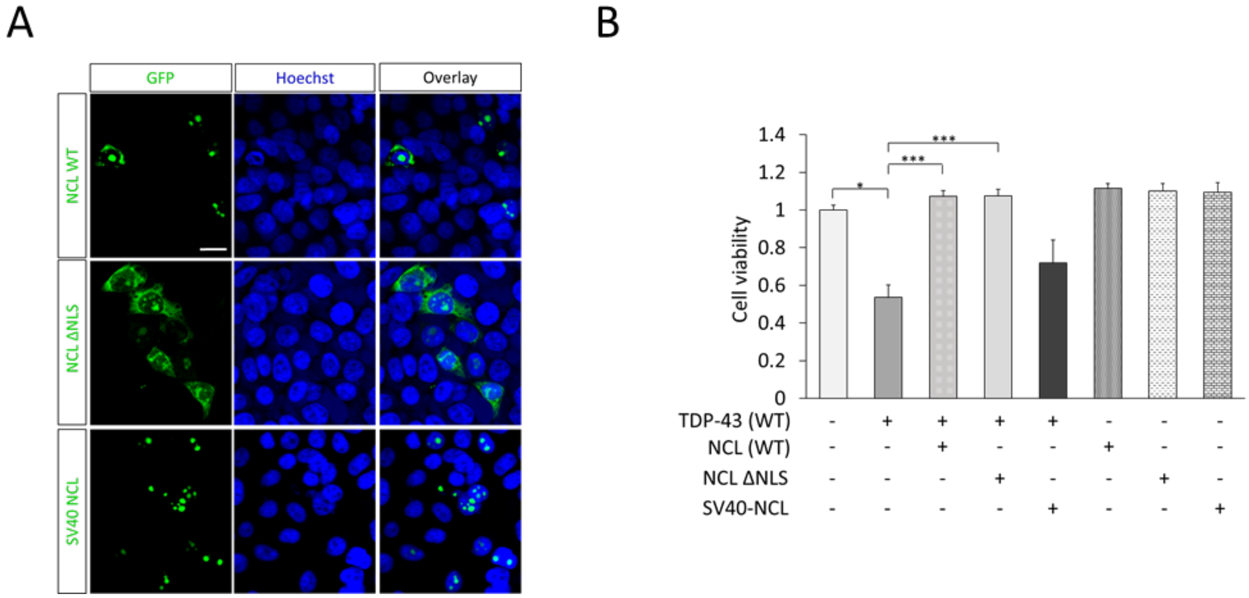

Supplementary Figure S9.

Mutant NCL lacking the nuclear localization sequence (ΔNLS) still rescues TDP-43 toxicity in human HEK293T cells, while the nuclear-targeted NCL mutant (SV40-NCL) does not.

A. Confocal microscopy analysis of HEK293T cells transfected with plasmids coding for the NCL-GFP chimera, either WT (NCL WT, upper panels), lacking the NLS sequence (NCL ΔNLS, middle panels) or fused to the strong nuclear localization SV40 sequence (SV40-NCL, lower panels). Cells were counterstained with the fluorescent Hoechst nuclear dye. Micrographs are representative of 3 biological replicates. Scale bar 20 µM.

B. Cell viability MTS assay on HEK293T cells co-transfected with plasmids coding for human WT TDP-43 fused to mKate2 and human GFP-fusion constructs of NCL, either WT, lacking the NLS sequence or fused to the SV40 sequence. As controls (-), cells were co-transfected with plasmids encoding mKate2 and/or GFP only. Data were normalized to the mean value of control samples (-/-/-/-). Data are mean ± SEM, n=7, * $p<0.05$, *** $p<0.001$ **** $p<0.0001$ Kruskal-Wallis test followed by a Dunn's post hoc test.

Supplementary Tables

Name	Relevant genotype	Parental genotype	Origin
IMX672	*MAT*a *ura3-52 trp1-289 leu2-3,112 his3Δ can1Δ::cas9-natNT2*		Mans et al. 2015
TDP 1C	*MAT*a *ura3-52 trp1-289 leu2-3,112 his3Δ::*Gal1-TDP-43-GFP-Cyc1 *can1Δ::cas9-natNT2*	IMX672	This study
TDP 2C	*MAT*a *ura3-52 trp1 Δ::* Gal1-TDP-43-GFP-Cyc1 *leu2-3,112 his3Δ:* Gal1-TDP-43-GFP-Cyc1 *can1Δ::cas9-natNT2*	TDP1C	This study
TDP 3C	*MAT*a *ura3-52 trp1 Δ::* Gal1-TDP-43-GFP-Cyc1 *leu2 Δ:* Gal1-TDP-43-EGFP-Cyc1 *his3Δ:* Gal1-TDP-43-EGFP-Cyc1 *can1Δ::cas9-natNT2*	TDP2C	This study
TDP 2C/NCL 2C	*MAT*a *ura3 Δ::* Gal1-NCL-mKate2-Cyc1 *trp1 Δ::* Gal1-TDP-43-GFP-Cyc1 *leu2 Δ:* Gal1-NCL-mKate2-Cyc1 *his3Δ:* Gal1-TDP-43-EGFP-Cyc1 *can1Δ::cas9-natNT2*	TDP2C	This study
Q331K TDP 1C	*MAT*a *ura3-52 trp1-289 leu2-3,112 his3Δ:* Gal1-TDP-43(Q331K)-GFP-Cyc1 *can1Δ::cas9-natNT2*	IMX672	This study
M337V TDP 1C	*MAT*a *ura3-52 trp1-289 leu2-3,112 his3Δ:* Gal1-TDP-43(M337V)-GFP-Cyc1 *can1Δ::cas9-natNT2*	IMX672	This study
Q331K TDP 2C	*MAT*a *ura3-52 trp1 Δ:* Gal1-TDP-43(Q331K)-GFP-Cyc1 *leu2-3,112 his3Δ:* Gal1-TDP43(Q331K)GFP-Cyc1 *can1Δ::cas9-natNT2*	Q331K TDP 1C	This study
M337V TDP 2C	*MAT*a *ura3-52 trp1 Δ:* Gal1-TDP-43(M337V)-GFP-Cyc1 *leu2-3,112 his3Δ:* Gal1-TDP43(M337V)GFP-Cyc1 *can1Δ::cas9-natNT2*	M337V TDP 1C	This study
ΔNSR1	*MAT*a *ura3-52 trp1-289 leu2-3,112 his3Δ can1Δ::cas9-natNT2 nsr1 Δ*	IMX672	This study

Supplementary Table 1 – *Saccharomyces cerevisiae* strains used in this study.

Name	Origin
Plasmids for S. cerevisiae	
pYES2	Thermo Fisher #V82520
mKate2-pYES2	(Vicario et al., 2017)
pYES2<His3>	This paper
mKate-pYES2<His3>	This paper
NCL-mKate-pYES2<His3>	This paper
□NLS-NCL-mKate2-pYES2<His3>	This paper
SV40-NCL-mKate2-pYES2<His3>	This paper
dsRED-NPM-pYES2<HIS3>	This paper
NCL-pYES2<His3>	This paper
NCL-pGADT7	This paper
pGADT7 AD	Takara-bio #630442
pRS426 Gal TDP43 GFP	ADDGENE #27467
pRS426 Gal TDP-43	This paper
pAG416-Gal-PR50	ADDGENE #84901
426Gal-FUS-YFP	ADDGENE #84901
NSR1-HA pYES2<His3>	This paper
pMEL10gHis3	This paper
pMEL16gLeu2	This paper
pMel14gTrp1	This paper
pMel16gUra3	This paper
Plasmids for mammalian cells	
mKate2-pCDNA3.1	(De Mario et al., 2016)
TDP43mKate2-pCDNA3.1	This paper
Q331K-TDP43mKate2-pCDNA3.1	This paper
pEGFPC1	Dbiosciences #6084-1
NCL-pEGFP	ADDGENE #28176
NCLΔNLS-pEGFP	This paper
SV40NCL-pEGFP	This paper
pCMV-DsRed-NPM	ADDGENE #34553

Supplementary Table 2 – List of plasmids used in this paper.

Primers	Sequence 5'3'	Aim
HIS3-REP-F	AATGTGATTTCTTCGAAGAATATACTAAAAAATGAGCAGG CAAGATAAACGAAGGCAAAGAGTACGGATTAGAAGCCGC CGAGC	Generation of repair cassette for the *HIS3* locus
HIS3-REP-R	GGTATACATATATACACATGTATATATATCGTATGCTGCAG CTTTAAATAATCGGTGTCAGCAAATTAAAGCCTTCGAGCGT CC	
TRP1-REP-F	AAAATAGTTCAGGCACTCCGAAATACTTGGTTGGCGTGTTT CGTAATCAACCTAAGTAGGAGTACGGATTAGAAGCCGCCG AGC	Generation of repair cassette for the *TRP1* locus
TRP1-REP-R	CACCAATAACGCCATTTAATCTAAGCGCATCACCAACATTT TCTGGCGTCAGTCCACCAGGCAAATTAAAGCCTTCGAGCGT CC	
LEU2-REP-F	GATGGTGTCGCTTGGGATAGTGAACAATACACCGTTCCAGA AGTGCAAAGAATCACAAGAAGTACGGATTAGAAGCCGCCG AGC	Generation of repair cassette for the *LEU2* locus
LEU2-REP-R	AAGTTCAATGACAATTTCAACATCATTGCAGCAGACAAGAT AGTGGCGATAGGGTTGACCGCAAATTAAAGCCTTCGAGCGT CC	
URA3-REP-F	TGCCCAGTATTCTTAACCCAACTGCACAGAACAAAAACCTG CAGGAAACGAAGATAAATCAGTACGGATTAGAAGCCGCCG AGC	Generation of repair cassette for the *URA3* locus
URA3-REP- R	TTAAATTGAAGCTCTAATTTGTGAGTTTAGTATACATGCATT TACTTATAATACAGTTTTGCAAATTAAAGCCTTCGAGCGTC C	

Supplementary Table 3 – Primers used for the generation of repair cassettes.

Supplementary Material

Primers	Sequence 5'3'
NSR1 repair F	CGAACTGTTAACCAATTTCGGATCACTCAACCCAGGCAGGATAAAATAAGTGAGAAAAT GAAATGAATTTCAATTTCAATTTTTTCTCTTTTTACGTTAA
NSR1 repair R	TTAACGTAAAAAGAGAAAAAATTGAAATTGAAATTCATTTCATTTTCTCACTTATTTTATC CTGCCTGGGTTGAGTGATCCGAAATTGGTTAACAGTTCG

Supplementary Table 4 – Primers used for dsDNA ΔNSR1 repair fragment generation.

Primers	Sequence 5'3'	
dgNSR1-F	CTGGGGGTAAGTGCCTGATG	Diagnostic primers for NSR1 deletion confirmation
dgNSR1 R	TGTTGCTAAACGCCATTGGC	
dgHIS3- F	GGAGTCACTGCCAGGTATCG	Diagnostic primers for confirmation of gene insertion in *his3* locus
dgHIS3-R	GAAACCACCGTTGCCGTAAC	
dgTRP1-F	TGGTGAAAGTTTGCGGCTTG	Diagnostic primers for confirmation of gene insertion in *trp1* locus
dgTRP1-R	CGGTTGTTTGCAAGACCGAG	
dgLEU2-F	GTGGGTGGTCCTAAATGGGG	Diagnostic primers for confirmation of gene insertion in *leu2* locus
dgLEU2-R	CTTTTTGTGTGGTGCCCTCC	
dgURA3-F	ACGAAGGAAGGAGCACAGAC	Diagnostic primers for confirmation of gene insertion in *ura3* locus
dgURA3-R	CCAGTACACCTTATCGGCCC	

Supplementary Table 5 – Diagnostic primers used for check integration/deletion correctness.

Primers	Sequence 5’-3’
HIS-PYES F	TACTTATAATACAGTCAATAGGCACTGCGGCATCAGAGCAGATTG
HIS-PYES R	CATCGATAAGCTAGCGAAAGGTGCATCTGTGCGGTATTTCACACC
pYESΔURA F	GCTAGCTTATCGATGATAAGCTG
pYESΔURA R	ACTGTATTATAAGTAAATGCATG
Ncl-mKate2pYES2 F	TACCGAGCTCGGATCCATGGTGAAGCTCGCGAAGG
Ncl-mKate2pYES2 R	CACCATCGATGGATCCCCTTCAAACTTCGTCTTCTTTCC
Delta KateNCL F	AGTTTGAATCGATGCACCACCACCAC
Delta KateNCL R	GCATCGATTCAAACTTCGTCTTCTTTCCTTG
Delta GFPTDP F	GGGGAATGTAACTCGAGTCATGTAATTAGTT
Delta GFPTDP R	CGAGTTACATTCCCCAGCCAGAAGA
NPM F	TACCGAGCTCGGATCCATGGAAGATTCGATGGACATGGACATGAGC
NPM R	ATGGATATCTGCAGAATTCCTACAGGAACAGGTGGTGGCGGCCCTCGGC
SV40-NCL F	TCCCGAGCCTCCAAAAAAGAAGAGAAAGGTCGAATTGGGTACCGAGCTCGGATCC A
SV40-NCL R	TTTGGAGGCTCGGGAATTAATTCCGCTTTATCCATAAGCTTAATATTCCCTATAGTG AGT
NCLΔNLS F	AAGCACCTGAAGGCACAGAACCGACTACG
NCLΔNLS R	TGCCTTCAGGTGCTTCTTTGACAGGC
SV40NCL GFP F	TTCCCGAGCCTCCAAAAAAGAAGAGAAAGGTCGAACTACCGGTCGCCACCATG
SV40NCL GFP R	TTGGAGGCTCGGGAATTAATTCCGCTTTATCCATCGCTAGCGGATCTGACGG
HA-NSR1PYES F	TACCGAGCTCGGATCCATGGAGTACCCATACGACGTACCAGATTACGCTGCTAAGA CTACTAAAGTAAAAGGTAACAAGAAGG
HA-NSR1PYES R	GATATCTGCAGAATTCTTAATCAAATGTTTTCTTTGAACCA
NCL-AD F	GGAGGCCACCAGTGAATTCATGGTGAAGCTCGCGAAGGCAGGTAAAAATCAAGG
NCL-AD R	CACCCGGGTGGAATTGCTATTCAAACTTCGTCTTCTTTCCTTGTGG
TDP-43 KATE F	CGCGGGCCCGGGATCCATGTCTGAATATATTCGGGTAACCG

TDP-43 KATE R	GGCGACCGGTGGATCCCCCATTCCCCAGCCAGAAGAC
TDP43 Q331K F	GCCCAGGCAGCACTAAAGAGCAGTTGGGG
TDP43 Q331K R	CCCCAACTGCTCTTTAGTGCTGCCTGGGC
TDP-43 M337V F	GAGCAGTTGGGGTATGGTGGGCATGTTAGCC
TDP-43 M337V R	GGCTAACATGCCCACCATACCCCAACTGCTC
pMEL NSR1 target R	TTCTAGCTCTAAAACATGTGTTCGAATTCCTTCTTGATCATTTATCTTTCACTGCGGAGA
pMELUra3 target R	TTCTAGCTCTAAAACAGGAATTACTGGAGTTAGTTGATCATTTATCTTTCACTGCGGAGA
pMELHis3 target R	TTCTAGCTCTAAAACTATACCTGTGTGGACGTTAAGATCATTTATCTTTCACTGCGGAGA
pMEL Leu2 target R	TTCTAGCTCTAAAACCCTAAATGGTATTATAATCAGATCATTTATCTTTCACTGCGGAGA
pMELTrp1 target R	TTCTAGCTCTAAAACAGTCAGAAATCGAGTTCCAAGATCATTTATCTTTCACTGCGGAGA
6006 F	GTTTTAGAGCTAGAAATAGCAAGTTAAAATAAGGCTAGTC

Supplementary Table 6 – Primers used for the generation of plasmids

UniProt Entry	Protein name	Peptide sequence	Precursor Ion (m/z)	Product 1 (m/z)	Product 2 (m/z)	Product 3 (m/z)	Product 4 (m/z)	Retention Time (min)	NCE
P53048	MAL11	NSQENLGNSDLGYK	769.858	853.4	480.3	367.2	202.1	10.8	27
		SGSFFNCFK	547.242	715.3	568.3	232.1	145.1	16.0	27
P0CX80	MTCU1	NNEQCQK	460.698	692.3	563.3	435.2	229.1	2.2	27
		SCSCPTGCNSDDK	744.263	993.4	795.3	335.1	248.1	5.3	27
P14904	AMPL	GVGVIGSHVDALTVK	484.612	246.2	570.3	214.1	157.1	13.9	27
		SALVDSTPLPVCR	707.872	1044.5	741.4	531.3	272.2	14.4	27
P23180	AIM17	LFQTLVNLQK	602.358	943.6	714.5	601.4	261.2	16.5	27
		GCYFDSDTFK	620.253	1022.4	859.4	712.3	218.1	14.0	27
P34227	PRX1	LIFTYPSTVGR	627.348	1027.5	779.4	616.3	227.2	16.2	27
		VIDALQLTDK	558.319	753.5	640.4	476.3	213.2	12.1	27
P37012	PGM2	YYNDVILHK	388.873	556.2	510.3	397.3	284.2	11.5	27
		IVDQLR	372.224	630.4	531.3	416.3	288.2	9.1	27
P38708	YHI0	DHVEGFAPEVAWVTR	571.618	756.3	632.4	561.3	253.1	17.4	27
		FSQYELK	457.735	767.4	680.4	552.3	235.1	11.2	27
P02829	HSP82	EEVQEIEELNK	680.336	874.5	745.4	261.2	259.1	12.6	27
		EILGDQVEK	515.774	788.4	675.3	618.3	243.1	10.5	27
P06738	PHSG	VLAVAYDFPVPGFK	761.919	1069.5	448.3	284.2	213.2	20.4	27
		VVFVADYNVSK	620.832	1042.5	796.4	346.2	199.1	14.2	27
P81449	ATPJ	LHPVVTPK	445.777	640.4	345.2	251.2	244.2	8.1	27
		VILNAVESLK	543.332	873.5	760.4	646.4	213.2	15.1	27
Q12305	RDL1	HDPNVVLVDVR	421.567	563.3	488.3	464.2	274.2	12.7	27
		SHPDAFALDPLEFEK	572.614	726.3	655.3	423.2	225.1	18.1	27
P14306	CPYI	WSEFCHLVECDLK	574.925	1013.5	876.4	763.4	535.3	16.7	27
		YVFLLYK	473.276	782.5	683.4	310.2	263.1	17.9	27
P30902	ATP7	VISSLR	337.713	575.4	462.3	375.2	213.2	8.6	27
		QLQVIESFEK	610.830	851.5	752.4	639.3	510.3	15.6	27
P41816	OYE3	DTNLFEPIK	538.785	860.5	633.4	357.2	217.1	15.7	27
		FFISNPDLVYR	685.859	1076.6	963.5	762.4	295.1	17.6	27
P05626	ATPF	IDSVSQLQNVAETTK	816.926	549.3	349.2	316.2	229.1	14.0	27
		VQSELGNPK	486.261	744.4	528.3	415.2	228.1	7.5	27
P22202	HSP74	ETAENFLGTEVK	669.333	907.5	793.4	646.4	533.3	14.5	27
		NSVSENNFK	519.746	837.4	738.3	301.2	202.1	8.1	27
P38804	SDO1L	GAEGELGAASK	495.249	732.4	546.3	433.2	129.1	7.7	27
		AQVENEFGK	511.251	822.4	723.3	204.1	200.1	9.2	27

Q04432	HSP31	FGWDEHSLAK	397.193	418.3	391.2	218.1	205.1	13.2	27
		DFLNGQDETDFK	714.818	1053.4	939.4	294.2	263.1	15.0	27
P15992	HSP26	LLGEGGLR	407.743	701.4	588.3	402.2	227.2	11.6	27
		ADYANGVLTLTVPK	731.401	1041.6	771.5	350.1	244.2	16.9	27
P22943	HSP12	DNAEGQGESLADQAR	780.848	1131.5	946.5	374.2	230.1	9.8	27
		LNDAVEYVSGR	611.807	809.4	710.3	581.3	343.2	12.2	27
P32874	HFA1	TPIEYLIELLETR	795.443	1072.6	959.5	760.4	405.2	22.8	27
		GFQYLYLAPK	600.327	956.5	591.4	428.3	315.2	16.6	27
P00358	G3P2	HIIVDGHK	306.843	456.2	364.2	341.2	251.2	5.7	27
		IATFQER	432.732	751.4	680.3	579.3	185.1	9.4	27
Q13148	TADBP_HUMAN	FTEYETQVK	572.780	896.4	767.4	604.3	249.1	10.2	27
		GISVHISNAEPK	626.338	895.5	758.4	645.3	258.1	10.6	27
P19338	NUCL_HUMAN	ALELTGLK	422.761	660.4	531.4	314.2	185.1	13.9	27
		GEGGFGGR	368.672	550.3	493.3	289.2	187.1	6.8	27

Supplementary Table 7 – Instrumental settings used for relative quantification of proteins in yeast cells. For each protein, the peptide sequence, mass-to-charge ratio (m/z) for the monitored precursor peptide and four fragmentation products (Product 1-4), expected retention time (RT) and normalized collision energy (NCE) are reported.

Protein name	UNIPROT symbol	TDP 1C/CENPK mean ratio	p-value	Biological process or molecular function GO annotation term
**ATP synthase subunit 4, mitochondrial**	**ATPF**	0.544	1.4E-02	ATP synthesis coupled proton transport
**ATP synthase subunit e, mitochondrial**	**ATPJ**	0.599	2.7E-02	
**ATP synthase subunit d, mitochondrial**	**ATP7**	0.621	4.0E-03	
Ornithine aminotransferase	CAR2	0.687	7.0E-03	Cellular amino acid biosynthetic process
D-3-phosphoglycerate dehydrogenase	SER3	0.708	4.4E-02	
**Thiosulfate:glutathione sulfurtransferase**	**RDL1**	0.578	2.30E-02	Thiosulfate sulfurtransferase activity
**Putative proline--tRNA ligase**	**YHI0 mitochondrialy**	0.595	3.60E-02	Prolyl-tRNA aminoacylation
V-type proton ATPase subunit B	VMA2	0.751	0.025	Carbohydrate derivative metabolic process
Glycine--tRNA ligase 1, mitochondrial	HOM3	0.766	0.003	Cellular amino acid biosynthetic process
Aspartokinase	GRS1	0.768	0.03	Glycyl-tRNA aminoacylation
Copper metallothionein 1-1	**MTCU1**	1.832	1.50E-02	Detoxification
Peroxiredoxin PRX1	**PRX1**	1.504	1.30E-02	Cell redox homeostasis
Thioredoxin-2	TRX2	1.472	6.00E-03	
Peroxiredoxin AHP1	AHP1	1.355	5.00E-03	
Glutaredoxin-1	GRX1	1.482	1.50E-02	
12 kDa heat shock protein	**HSP12**	1.567	5.00E-03	Cell response to heat
Heat shock protein 104	HSP104	1.404	8.0E-03	
Heat shock protein SSA1	HSP71	1.449	3.0E-03	Protein folding-refolding
Heat shock protein 78, mitochondrial	HSP78	1.343	1.6E-02	
ATP-dependent molecular chaperone HSP82	**HSP82**	1.901	3.00E-03	
Heat shock protein 42	HSP42	1.464	3.0E-02	
Glutathione-independent glyoxalase HSP31	**HSP31**	1.520	2.0E-03	Response to stress
Heat shock protein SSA4	**HSP74**	2.024	1.0E-03	
NADPH dehydrogenase 3	**OYE3**	2.189	2.3E-02	Oxidation-reduction process
Heat shock protein 26	**HSP26**	1.785	2.2E-02	Protein folding
Probable oxidoreductase AIM17	**AIM17**	1.608	1.50E-02	Oxidation-reduction process
Ubiquitin-activating enzyme E1	UBA1	1.393	2.60E-02	Response to stress
Protein MMF	MMF1	1.482	3.50E-02	Cellular amino acid biosynthetic
Phosphoribosylaminoimidazole-succinocarboxamide	ADE1	1.48	4.80E-02	Carbohydrate derivative metabolic process
Acetyl-CoA carboxylase	**HFA1**	3.009	7.00E-03	
Phosphoglucomutase 2	**PGM2**	1.585	5.0E-03	Cellular carbohydrate metabolism
Alpha-glucosidase MAL32	MAL32	1.495	1.30E-02	
General alpha-glucoside permease	**MAL11**	2.574	1.00E-02	
Glycogen phosphorylase	**PHSG**	1.602	2.60E-02	
Restriction of telomere capping protein 3	**SDO1L**	1.55	3.60E-04	Ribosome biogenesis, rRNA processing
Vacuolar aminopeptidase 1	**AMPL**	1.566	1.70E-02	Cytoplasm to vacuole transport
Carboxypeptidase Y inhibitor	**CPYI**	1.589	2.00E-03	Regulation of proteolysis
Meiotic sister chromatid recombination protein 1	MSC1	1.450	1.00E-03	Reciprocal meiotic recombination

Supplementary Table 8 – Down- and up-regulated proteins in the TDP 1C yeast strain. Among the 631 proteins identified and quantified in both native CENPK and TDP 1C yeast strains, only proteins whose expression ratio (TDP 1C vs CENPK cells) was <0.77 (white lines) and >1.33 (light grey lines) (p-value <0.05, two-tailed t-test) are reported. Protein name and UNIPROT symbol are reported in the first two columns, while the last column reports the UniProtKB-assigned biological process or molecular function Gene Ontology (GO) annotation term. PRM (parallel reaction monitoring) validation study was performed only for proteins reported in bold.

GO biological process	p-value	PROTEIN NAME
**cellular response to oxidative stress**	1.40E-04	PRX1|GRX1|HSP31|HSP12|TRX2|MTCU1|HSP104|AHP1
**cellular oxidant detoxification**	5.30E-04	PRX1|GRX1|TRX2|MTCU1|AHP1
**cellular response to heat**	0.0014	HSP26|HSP78|HSP74|HSP12|HSP104
**protein folding**	0.0023	HSP71|HSP26|HSP78|HSP74|HSP104|HSP82
**cell redox homeostasis**	0.0029	PRX1|GRX1|TRX2|AHP1
**protein refolding**	0.0046	HSP71|HSP78|HSP82
**oxidation-reduction process**	0.0046	PRX1|GRX1|SER3|TRX2|AIM17|MTCU1|AHP1|PGM2|OYE3|PHSG
**stress granule disassembly**	0.0048	HSP71|HSP104
**response to stress**	0.0051	PRX1|HSP26|GRX1|HSP78|HSP31|HSP74|HSP12|TRX2|MTCU1|UBA1|HSP104|AHP1|HSP82
**response to abiotic stimulus**	0.0073	HSP26|HSP78|HSP74|HSP12|HSP104|HSP82
**proton transmembrane transport**	0.006	VMA2|ATPJ|MAL11|ATP7|ATPF
**response to metal ion**	0.0116	PRX1|MTCU1|AHP1
**carbohydrate derivative metabolic process**	0.0124	ADE1|VMA2|ATPJ|TRX2|ATP7|PGM2|HFA1|ATPF
**cellular response to reactive oxygen species**	0.0362	MTCU1|HSP104
**cellular amino acid biosynthetic process**	0.0389	HOM3|SER3|MMF1|CAR2

Supplementary Table 9 – The STRING analysis of the most deregulated 38 proteins reported in Fig. 7A also provided a gene ontology (GO) enrichment of the biological processes in which such proteins are involved. The significance of the GO biological processes was determined by false discovery rate correction (p-value < 0.05).

Supplementary Methods

Yeast strains construction.

S. cerevisiae strain CEN.PK IMX672 (Euroscarf) (Supplementary Table 1) was used as the background strain for all genetic manipulations by exploiting the CRISPR/Cas9 technique, as described in Mans et al. 2015.

The P_{Gal1}-TDP-43-GFP-CYC1TT, either carrying WT or mutated (Q331K and M337V) TDP-43 [TDP], or the P_{Gal1}-NCL-mKate2-CYC1TT [NCL] repair cassettes were PCR-amplified using as template the pRS246GalTDP43 (encoding for WT or ALS-related mutants Q331K and M337V) or Ncl-mKate2-pYES2 plasmids, respectively. Different pairs of forward (F) and reverse (R) primers (Supplementary Table 3) were used depending on the different locus in which the cassette were integrated.

In vivo integration of TDP or NCL cassettes in the chromosome loci *his3*, *trp1*, *leu2* or *ura3* was achieved by co-transforming CEN.PK IMX672 yeast strain with 1 µg of PCR-amplified repair cassette (TDP or NCL) and 500 ng of the gRNA expression vectors (pMEL10gHis3, pMEL14gTrp1, pMEL16gLeu2, pMEL16gUra3) (Supplementary Table 2), targeting to the chosen locus. Yeast transformants were selected using the auxotrophic markers of pMEL plasmids, hence by plating on the appropriate selective SD plates.

The deletion of NSR1 gene was achieved via transformation of CEN.PK IMX672 yeast strain with the pMEL10gNSR1 plasmid (Supplementary Table 2), targeting to the NSR1 locus, and the 120 bp dsDNA NSR1Δ repair fragment. To obtain dsDNA, the two complementary single-stranded oligos, ΔNSR1 repair F and R primers (Supplementary Table 4), were mixed in a 1:1 ratio, heated to 95°C for 5 min and cooled down to room temperature. Transformants were selected on SD-URA selective plates.

In all cases, gene deletions and integrations were confirmed by colony PCR on randomly picked colonies, using the diagnostic primers listed in Supplementary Table 5. After confirmation of the relevant genotype, pMEL plasmids were removed as described in Mans et al. 2015.

All primers were designed using the online webservice Yeastriction v0.1 that is available at https://github.com/hillstub/Yeastriction.

Plasmids propagation and construction.

E. coli strains StellaR [(F-, endA1, supE44, thi-1, recA1, relA1, gyrA96, phoA, Φ80d lacZΔ M15, Δ(lacZYA-argF) U169, Δ(mrr-hsdRMS-mcrBC), ΔmcrA, λ-)Clontech] and Top10 [{F-mcrA Δ(mrr-hsdRMS-mcrBC) ϕ80lacZΔM15 ΔlacX74 recA1 araD139 Δ(ara-leu) 7697 galU galK rpsL (StrR) endA1 nupG λ- }, Invitrogen] were used as a host for cloning procedures and plasmid propagation. *E. coli* cells were grown at 37°C in liquid or solid (by adding agar 2% (w/v)) Luria-Bertani (LB) medium (Tryptone 1% (w/v), Yeast extract 0.5% (w/v) NaCl. 1% (w/v)) containing ampicillin (100 µg/ml) or kanamycin 50 (µg/ml) (both from Sigma-Aldrich) for the selection of transformants.

All new generated plasmids (Supplementary Table 2) were obtained using the In-Fusion HD Cloning Kit (Takara-bio). Briefly, PCR products and digested (or PCR-amplified) vectors were spin-column purified using the GenElute™ PCR Clean-Up Kit (Sigma-Aldrich) and subjected to In-Fusion Cloning Procedure following the manufacturer's instruction. In all cases, Stellar *E. coli* competent cells were then transformed with the In-Fusion reaction mixture. Plasmids were isolated from grown transformants onto selective LB plates by standard methods and verified both by restriction digestion and by sequencing.

All primers (reported in Supplementary Table 6) were designed in order to obtained PCR products with 15 base pairs complementary extentions necessary for the *in vitro* recombination event.

The pYES2(HIS3) and mKate2-pYES2(HIS3) plasmids were generated by substituting the *URA3* cassettes of the plasmids pYES2 and pYES2-mKate2 with the HIS3 auxotrophic marker. Briefly, the HIS3 cassette was amplified from pMEL16 vector (Euroscarf) using HIS-PYES F and R primers, while the pYES2 or pYES2-mKate2 plasmids were amplified using the pYESΔURA F and R primers allowing the amplification of the backbone without the URA3 sequence. The PCR products were then subjected to In-Fusion cloning procedure as described above.
To express the fusion protein NCL-mKate2 in yeast, the NCL-mKate2-pYES2(HIS3) was generated by cloning the PCR-amplified NCL cDNA - using NclpEGFP plasmid as template and Ncl-mKate2-PYES2 F and R primers - in the BamHI linearized mKate2-pYes2(HIS3) vector.
The expression in yeast of native TDP-43 sequence was allowed by removing the GFP ORF sequence from the pRS426GalTDP43GFP plasmid through its PCR-amplification using DeltaGFP-TDP F and R primers. To express the native NCL sequence in yeast, the NCL-pYES2(HIS3) plasmid was constructed by PCR-amplification of NCL-mKate2-pYES2(HIS3) vector with DeltaKate-NCL F and R primers. These primers were designed to allow the removal of the sequence encoding for mKate2. In both cases the PCR products were then processed using the In-Fusion protocol.
The ΔNLS-NCL-mKate2-pYES2(HIS3) plasmid, in which the bipartite nuclear localization signal (NLS) (KRKKEMANKSAPEAKKKK) was removed, and the SV40-NCL-mKate2-pYES2(HIS3) plasmid, encoding for a SV40-NCL-mKate2 chimera, were generated by PCR-amplification of the NCL-mKate2-pYES2(HIS3) plasmid using NclΔNLS and SV40-Ncl F and R primers, respectively. PCR products were then subjected to In-Fusion cloning protocol.
For dsRED-NPM-pYES2(HIS3) plasmid creation, dsRED-NPM chimera was PCR-amplified using pCMV-DsRed-NPM as template and NPM F and R primers, and cloned in the BamHI-EcoRI digested pYES2(HIS3) plasmid.
Conversely, the PCR-amplified NCL ORF, using NCLpEGFP plasmid as template, and the NCL-AD F and R primers, was directly cloned in the EcoRI/BamHI linearized pGADT7 AD vector, to obtain the NCL-pGADT7 AD plasmid.
To create HA-NSR1-pYES2(HIS3), NSR1 ORF gene was amplified from CENPK genomic DNA using HA-NSR1PYES F (in which the nucleotide sequence of HA tag was inserted) and R primers, and cloned in BamHI/EcoRI linearized pYES2(HIS3) plasmid.
To clone TDP-43 in mKate2-PCDNA3.1 vector, TDP-43 coding sequence was PCR-amplified with TDP-43 KATE F and R primers using TDP-43-pRSgal416 plasmid as template. BamHI linearized mKate2-PCDNA3.1 vector and PCR amplified TDP-43 cDNA was then subjected to In-Fusion cloning protocol.
SV40-NCLpEGFP and NCLΔNLSpEGFP were generated by PCR-amplification of the NCLpEGFP plasmid using the SV40-NCL GFP (F and R) or NCLΔNLS (F and R) primers, respectively, and subjecting PCR products to the *In-Fusion*® cloning protocol.
Q331K point mutation were introduced by PCR in both TDP-43-mKate2-pCDNA3.1 and TDP-43pRS426gal plasmids, while M337V point mutation only in the latest, using the QuikChange Site-Directed Mutagenesis kit (Stratagene). Mutagenic oligonucleotides (Q331K F and R; M337V F and R) were designed using the web-based QuikChange Primer Design Program available online.

pMEL10, pMEL14, pMEL16, pMEL17 plasmids (Euroscarf) were used as backbone for constructing the single gRNA plasmids (pMEL10gHis3, pMEL14gTrp1, pMEL16gLeu2, pMEL16gUra3 and

pMEL10gNSR1). PCR was performed using the 6006 F primer (Mans et al. 2015) and a target R primer, specific for the desired locus (Supplementary Table 6). Here again, PCR products were processed using the In-Fusion Cloning Procedure following the manufacturer's instruction.

Supplementary References

De Mario, A., Scarlatti, C., Costiniti, V., Primerano, S., Lopreiato, R., Calì, T., Brini, M., Giacomello, M., and Carafoli, E. (2016). Calcium Handling by Endoplasmic Reticulum and Mitochondria in a Cell Model of Huntington's Disease. PLoS Curr. *8*.

Vicario, M., Calì, T., Cieri, D., Vallese, F., Bortolotto, R., Lopreiato, R., Zonta, F., Nardella, M., Micalizzi, A., Lefeber, D.J., et al. (2017). A novel PMCA3 mutation in an ataxic patient with hypomorphic phosphomannomutase 2 (PMM2) heterozygote mutations: Biochemical characterization of the pump defect. Biochim. Biophys. Acta - Mol. Basis Dis. *1863*, 3303–3312.
